# Impact of baseline symptoms and health status on COPD exacerbations in the FLAME study

**DOI:** 10.1186/s12931-020-01354-8

**Published:** 2020-04-22

**Authors:** Alexander J. Mackay, Konstantinos Kostikas, Nicolas Roche, Stefan-Marian Frent, Petter Olsson, Pascal Pfister, Pritam Gupta, Francesco Patalano, Donald Banerji, Jadwiga A. Wedzicha

**Affiliations:** 1 National Heart and Lung Institute, Imperial College London, London, UK; 2 Respiratory Medicine Department, University of Ioannina Medical School, Ioannina, Greece; 3 Pneumologie Hôpital Cochin (APHP), Université Paris Descartes (EA2511), Paris, France; 4 Department of Pulmonology, University of Medicine and Pharmacy Timisoara, Timisoara, Romania; 5 Novartis Sverige AB, Kista, Sweden; 6 Novartis Pharma AG, Basel, Switzerland; 7 Novartis Healthcare Pvt. Ltd., Hyderabad, India; 8 Novartis Pharmaceuticals Corporation, East Hanover, NJ USA

**Keywords:** Indacaterol/glycopyrronium, Dyspnoea, Bronchitis, Health status, COPD burden

## Abstract

**Background:**

COPD is a heterogeneous disease and patients may respond differently to therapies depending on baseline symptom burden.

**Methods:**

This post-hoc analysis from the 52-week FLAME study investigated the impact of baseline symptom burden in terms of health status, dyspnoea, bronchitis status, eosinophil levels and smoking status on the subsequent risk of moderate or severe exacerbations. Health status was measured by St. George’s Respiratory Questionnaire (SGRQ) score (higher ≥46.6 and lower < 46.6) and COPD Assessment Test (CAT) score (higher ≥17 and lower < 17); dyspnoea and bronchitis were assessed via an electronic diary (eDiary). Differential response to once-daily indacaterol/glycopyrronium (IND/GLY) 110/50 μg versus twice-daily salmeterol/fluticasone (SFC) 50/500 μg was assessed.

**Results:**

Data from 3354 patients was analysed. The risk of exacerbations was lower in patients who had less severe health impairment (rate ratio [RR] [95% CI]): SGRQ-C, (0.88 [0.78, 0.99]); CAT, 0.85 [0.75, 0.96]) and lower dyspnoea (0.79 [0.69, 0.90]) at baseline versus those with more severe health impairment and higher dyspnoea, respectively. Compared with SFC, IND/GLY led to better prevention of moderate-to-severe exacerbations in the majority of groups studied.

**Conclusion:**

Patients with more severe health status impairment and greater symptom burden at baseline subsequently experienced more exacerbations in the FLAME study. IND/GLY was overall more effective in preventing exacerbations versus SFC, regardless of baseline symptom burden. Our results suggest that future studies on novel exacerbation therapies should consider targeting patients with higher symptom burden at baseline.

**Clinical trial identifier:**

NCT01782326.

## Background

Chronic obstructive pulmonary disease (COPD) is heterogeneous and characterised by persistent airflow limitation and worsening of symptoms. Current treatment goals include reducing symptom burden, improving quality of life and lowering the risk of future exacerbations [1]. Previous reports suggest that impairments in health status are associated with increased risk of exacerbation-related morbidity [1].

In clinical trials, health status of a COPD patient is assessed using validated questionnaires such as the St. George’s Respiratory Questionnaire for COPD (SGRQ-C) and COPD Assessment Test (CAT) [2, 3]. Patients with elevated CAT [4] and SGRQ-C [5] scores experience greater exacerbation frequency than patients with lower CAT and SGRQ scores. While all COPD symptoms can predict long-term treatment response, breathlessness (dyspnoea) is one of the best predictors [6]. Dyspnoea is a cardinal feature and the key symptom of COPD, causing distress that contributes significantly to the disease burden [7–9]. Dyspnoea grade is closely linked to exacerbation risk [9], and severity of dyspnoea is also a significant predictor of mortality [7]. Consequently, as dyspnoea severity increases, so do the healthcare costs of managing patients with COPD [8, 10].

The presence of bronchitis is common in patients with COPD, which is characterised by chronic cough and excessive sputum production leading to a higher symptom burden [11, 12]. Higher symptom burden and chronic mucus hypersecretion are also associated with a higher risk of accelerated decline in lung function, exacerbations and hospitalisations [13–15]. An electronic diary (eDiary) enables the evaluation of individual and collective symptoms, including dyspnoea and bronchitis symptoms. Recent reports have indicated that daily symptoms captured by an eDiary may predict long-term response to bronchodilators [16, 17].

Earlier reports showed positive associations between blood eosinophil levels and the risk of exacerbation. Patients with high blood eosinophil levels (≥300 cells/μL) experience increased rates of exacerbations compared with patients with low eosinophil levels (< 300 cells/μL) [18]. However, eosinophil levels fluctuate over time [19] and their role as a biomarker for treatment decisions in COPD needs to be further refined. Smoking is one of the major factors that accelerate the deterioration of health status in patients with COPD. Studies have shown that smoking is associated with higher rates of exacerbations in patients with COPD leading to higher healthcare resource utilization [20–22].

Although, previous reports suggest an association between symptom burden in terms of health status impairment, dyspnoea, bronchitis, blood eosinophil levels and smoking status on the future risk of exacerbation, data from clinical trials on this association are scarce. Furthermore, differences in treatment response to dual bronchodilation therapy by baseline health status, symptom, disease and clinical characteristics along with smoking status would support the appropriate targeting of different patient phenotypes and enable personalised therapy. In this post hoc analysis from the landmark FLAME study [23], we investigated the impact of baseline health status, symptom severity, key clinical characteristics, including dyspnoea and bronchitis, eosinophil levels and smoking status on the risk of exacerbations and response to treatment in COPD patients.

## Methods

### Study design and patients

Data from the FLAME study were used to perform this analysis. In the FLAME study, patients with ≥1 exacerbation in the past year were randomised to indacaterol/glycopyrronium (IND/GLY) 110/50 μg once daily or salmeterol/fluticasone (SFC) 50/500 μg twice daily [23] for 52 weeks (ClinicalTrials.gov number, NCT01782326).

Patients included were aged ≥40 years, with symptomatic COPD (modified Medical Research Council [mMRC] scale ≥2), a post-bronchodilator forced expiratory volume in 1 s (FEV_1_) of ≥25 to < 60% of the predicted value, and a post-bronchodilator FEV_1_/forced vital capacity < 0.70.

Patients were excluded if they had exacerbations requiring treatment with antibiotics and/or systemic corticosteroids and/or hospitalisation in the 6 weeks prior to screening. Patients with any history of asthma or a blood eosinophil count > 600/mm^3^ at the start of the run-in period were also excluded. The majority of the patients included in the FLAME study were from group D according to global initiative for chronic obstructive lung disease (GOLD) 2016 criteria [23, 24]. The study design was presented in the primary publication [23] and in Supplementary Figure S1.

The FLAME study protocol and all amendments were reviewed by an Independent Ethics Committee or Institutional Review Board. The study was conducted based on the Declaration of Helsinki and Good Clinical Practice guidelines. Permission was obtained for use of SGRQ-C and the questionnaire was used without modification. The SGRQ-C permission document can be found in Supplementary section S1.

### Assessments

In this post hoc analysis, regardless of treatment, patients from the full analysis set of the FLAME study were divided into groups based on baseline symptoms, presence of disease, clinical characteristics and smoking status.

CAT and SGRQ were used to measure health status; patients were divided into higher or lower health status impairment groups with split at median based on CAT and SGRQ levels obtained at Day 1. Lower scores denoted better health status and vice versa (lower scores, CAT < 17 and SGRQ < 46.6; higher scores, CAT ≥17 and SGRQ ≥46.6). Higher and lower dyspnoea burden at baseline were measured via eDiary: “During what activities did you first feel breathless in the last 24 hours?” on a scale of 0 to 3 (0 = Never or only when running; 1 = When walking uphill or upstairs; 2 = When walking on flat ground; 3 = At rest). Patients were divided into higher or lower dyspnoea groups with split at median based on daily highest dyspnoea scores averaged over the run-in period (approximately 28 days), including the morning assessments at Day 1.

Bronchitis was evaluated based on patients’ response to a specific question in the Exacerbations and Symptoms in COPD (ESCO) eDiary: “How much sputum did you produce in the past 12 hours?” on a scale of 0 to 3 (0 = none; 1 = < 5 mL/1 teaspoon [tsp]; 2 = 5–25 mL/1–5 tsp; 3 = > 25 mL/5 tsp). Bronchitis and non-bronchitis patients were defined as those with daily highest sputum volume score of ≥1 (bronchitis) or < 1 (non-bronchitis), for ≥50% of the time during the run-in period including Day 1.

Patients were further stratified into lower (< 300 cells/μL) and higher (≥300 cells/μL) blood eosinophil groups. The impact of the blood eosinophil levels on the future rate of exacerbations was evaluated in patients with higher or lower eosinophil levels.

At baseline patients were grouped based on their smoking status (current smoker vs ex-smokers). The risk of moderate or severe exacerbations were estimated in smokers compared with ex-smokers.

Further, differential response to IND/GLY and SFC in terms of future risk of moderate or severe exacerbation was measured based on patients’ baseline health status, dyspnoea, bronchitis status, blood eosinophil levels and smoking status.

Exacerbations were defined symptomatically using the eDiary data according to the criteria of Anthonisen et al. [25] and based on healthcare resource utilisation [26]. In brief, COPD exacerbations were defined as a worsening of ≥2 major symptoms (dyspnoea, sputum purulence or sputum volume) or as a worsening of any one major symptom and a minor symptom (sore throat, colds [nasal discharge and/or nasal congestion], fever without other cause, cough or wheeze) for at least 2 consecutive days. Only moderate or severe healthcare resource utilisation events were included in this analysis. If an exacerbation required treatment with oral corticosteroids and/or antibiotics, it was considered as moderate, whereas, an exacerbation leading to hospitalisation within 7 days from the onset was considered as severe. Hypothetical total symptom burden (measured using the eDiary data) at the onset and during an exacerbation is graphically represented in Supplementary Figure S2.

### Statistical analyses

A generalised linear model with negative binomial distribution was used to compare the rate of moderate or severe exacerbations based on the health status impairment, dyspnoea, blood eosinophil levels, bronchitis and smoking status. Rate of exacerbations were also analyzed within these groups with respect to treatments, IND/GLY and SFC. The offset variable log exposure time (in years) was used in the model which included fixed effects of treatment, baseline total symptom score, baseline COPD exacerbation history during the past 12 months of the study, smoking status (except for the comparison between current and ex-smokers where smoking status was considered as a subgroup and not a fixed effect), inhaled corticosteroid use, airflow limitation, age, sex and region for analysing within higher or lower symptom groups. The symptom groups considered were: bronchitis or non-bronchitis, higher or lower dyspnoea, higher or lower SGRQ and CAT scores, higher or lower blood eosinophil levels and with or without smoking. For overall comparisons between the two symptom groups, irrespective of the treatment received, the groups (high or low health status impairments/bronchitis or non-bronchitis) were considered as fixed effects instead of the treatments. Moderate or severe COPD exacerbations starting between the first dose and 1 day after last treatment dose were included. COPD exacerbations occurring within 7 days of each other were collapsed as one event. A linear mixed ANCOVA model was used to analyse the peak symptom score for each patient based on eDiary symptoms (except rescue medications) averaged over the period of moderate or severe exacerbations, using the above mentioned variables with the exception of not including COPD exacerbation history as a covariate and assuming a random effect of center nested within region.

## Results

### Baseline characteristics

In total, 3354 patients were evaluated in this analysis. In brief, the mean age of the overall population was approximately 65 years and 75% of patients belonged to GOLD (2016) group D [23, 24]. Patients’ baseline demographics and clinical characteristics are presented in Table 1.

**Table 1:** Baseline demographics and clinical characteristics (full analysis set)

Characteristic	IND/GLY ***N*** = 1675	SFC ***N*** = 1679	Total ***N*** = 3354
Age, years	64.6 ± 7.89	64.5 ± 7.70	64.6 ± 7.79
Men, n (%)	1295 (77.3)	1255 (74.7)	2550 (76.0)
Current smoker, n (%)	660 (39.4)	667 (39.7)	1327 (39.6)
Duration of COPD, years	7.2 ± 5.32	7.3 ± 5.44	7.3 ± 5.38
Number of COPD exacerbations in the previous year, n (%)			
0	1 (0.1)	1 (0.1)	2 (0.1)
1	1350 (80.6)	1352 (80.5)	2702 (80.6)
≥ 2	324 (19.3)	325 (19.4)	649 (19.4)
Post-bronchodilator FEV_1_, % predicted	44.0 ± 9.47	44.1 ± 9.43	44.1 ± 9.45
SGRQ-C total score	47.3 ± 15.83	47.2 ± 15.86	47.3 ± 15.84
CAT score	16.9 ± 7.06	16.6 ± 6.97	16.7 ± 7.02
eDiary total score	6.6 ± 2.93	6.5 ± 2.87	6.6 ± 2.90

Data presented as mean ± SD unless otherwise specified

CAT, COPD assessment test; eDiary, electronic diary; FEV_1_, forced expiratory volume in 1 s; IND/GLY, indacaterol/glycopyrronium 110/50 μg once daily; SFC, salmeterol/fluticasone 50/500 μg twice daily; SGRQ-C, St. George’s Respiratory Questionnaire for COPD.

### Impact of baseline health status measured via CAT and SGRQ-C scores on moderate/severe exacerbation rate

**Fig. 1:**
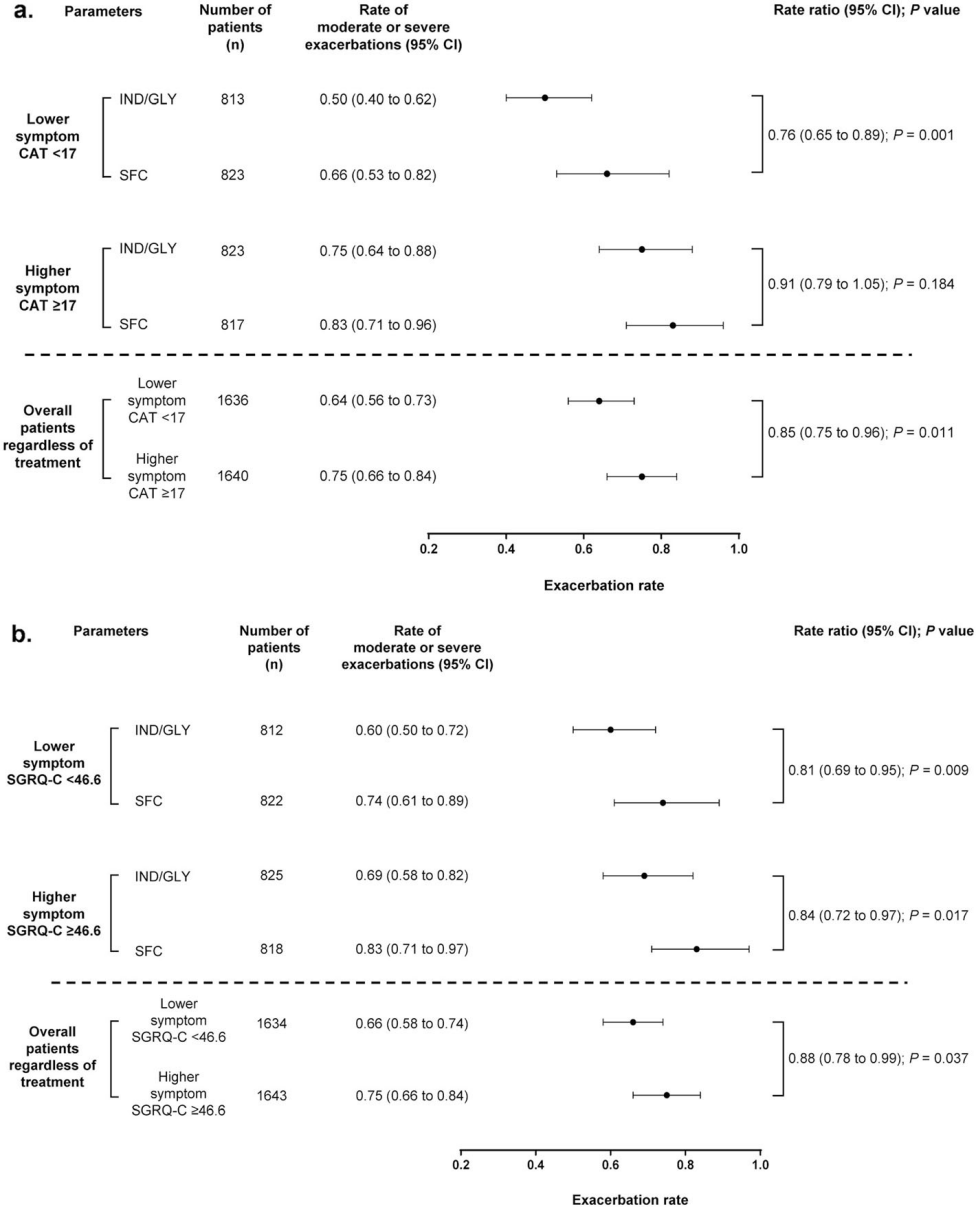
Annualised rate of COPD exacerbations by baseline. (a) CAT and (b) SGRQ-C total scores. n, number of patients assessed in this analysis. CAT, COPD Assessment Test; CI, confidence interval; IND/GLY, indacaterol/glycopyrronium 110/50 μg once daily; SFC, salmeterol/fluticasone 50/500 μg twice daily; SGRQ-C, St. George’s Respiratory Questionnaire for COPD

Patients with lower baseline CAT (< 17) and SGRQ-C (< 46.6) scores had lower exacerbation rates (rate ratio [RR] for CAT: 0.85; *P* = 0.011 and SGRQ-C: 0.88; *P* = 0.037), regardless of treatment received. Patients treated with IND/GLY and who had lower CAT score, experienced fewer exacerbations than those receiving SFC. However, both IND/GLY and SFC showed comparable efficacy in patients with higher CAT score. When baseline symptoms were measured via SGRQ-C scores, IND/GLY showed greater reduction in exacerbations vs SFC in both patients with higher and lower baseline symptom burden (Fig. 1a and b). Patients with less severe health status impairment at baseline had a lower mean percentage of days on exacerbation. Symptom severity during exacerbations (measured by eDiary) was lower in patients with lower baseline SGRQ scores; however, severity was comparable when we measured baseline health status via CAT scores (data presented in Supplementary Figures S3a and S3b).

### Impact of baseline dyspnoea on the rates and characteristics of moderate or severe exacerbations

**Fig. 2:**
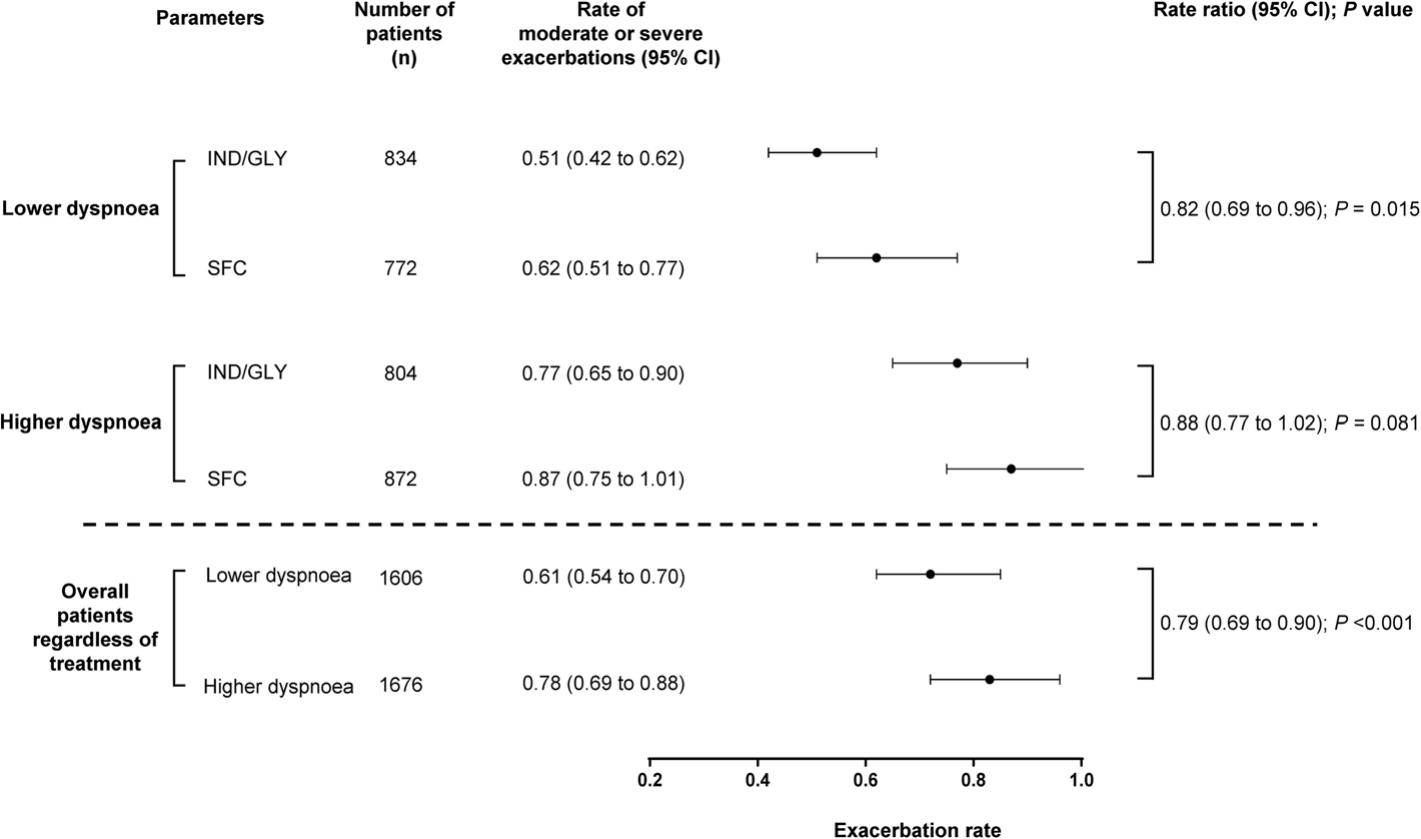
Annualised rate of COPD exacerbations by baseline dyspnoea level measured via eDiary. n, number of patients assessed in this analysis. Dyspnoea was measured via eDiary based on median split of daily highest dyspnoea scores averaged over the run-in period. CI, confidence interval; COPD, chronic obstructive pulmonary disease; eDiary, electronic diary; IND/GLY, indacaterol/glycopyrronium 110/50 μg once daily; SFC, salmeterol/fluticasone 50/500 μg twice daily

Patients with lower baseline dyspnoea had a lower annualised exacerbation rate than those with higher dyspnoea (RR, 0.79; *P* < 0.001). In patients with lower dyspnoea scores, reduction of exacerbation frequency was greater with IND/GLY versus SFC. However, in patients with higher dyspnoea scores, efficacy of IND/GLY was comparable with SFC (Fig. 2). Peak symptom severity and exacerbation duration (measured by percentage of days on exacerbation) were lower in patients with lower baseline dyspnoea (data presented in Supplementary Figures S4a and S4b).

### Impact of baseline bronchitis status on the rates and characteristics of moderate/severe exacerbations

**Fig. 3:**
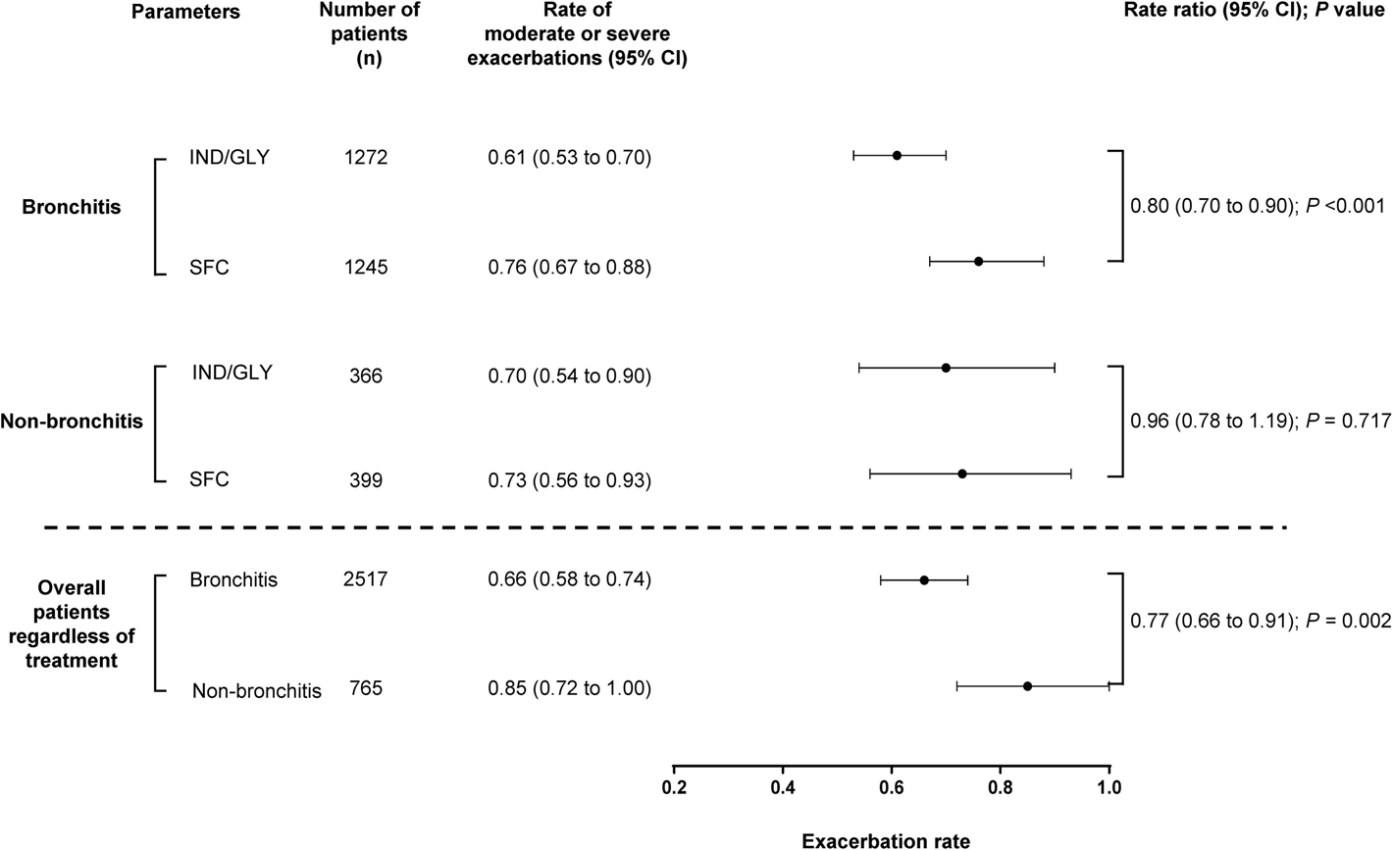
Annualised rate of COPD exacerbations by baseline bronchitis status measured via eDiary. n, number of patients assessed in this analysis. Bronchitis was evaluated based on patients’ response to a specific question in the eDiary. Bronchitis and non-bronchitis patients were defined as those with daily highest sputum volume score of ≥1 (bronchitis) or < 1 (non-bronchitis) for ≥50% of the time during the run-in period. CI, confidence interval; COPD, chronic obstructive pulmonary disease; eDiary, electronic diary; IND/GLY, indacaterol/glycopyrronium 110/50 μg once daily; SFC, salmeterol/fluticasone 50/500 μg twice daily

Of the 3354 patients included in the FLAME study (full analysis set), 76% with available information met the criteria for bronchitis. Patients with bronchitis experienced a lower rate of exacerbations (RR, 0.77; *P* < 0.002), regardless of treatment received. Patients with bronchitis treated with IND/GLY had a lower annualised exacerbation rate than patients who received SFC (Fig. 3). In patients with bronchitis who experienced exacerbations, there was no difference in terms of intensity of symptoms during exacerbation between treatment groups; however, IND/GLY lowered mean percentage of days on exacerbation versus SFC. Further, both treatments had a comparable effect on symptom burden during exacerbation, regardless of bronchitis status (data are presented in Supplementary Figures S5a and S5b).

### Impact of other baseline symptoms on the rates and characteristics of moderate/severe exacerbations

Other individual baseline symptoms had no impact on the rate of exacerbations. Results for annualised rate of COPD exacerbations by baseline total symptom burden measured via eDiary are presented in the Supplementary Figure S6, mean percentage of days on exacerbation in Figure S7a and mean peak symptom scores during exacerbations in Figure S7b.

### Impact of baseline eosinophil levels on the rates and characteristics of moderate/severe exacerbations

There was no impact of baseline blood eosinophil levels on the rate of exacerbation (RR, 1.03 [0.91 to 1.18]; *P* = 0.624) and total symptom burden (Supplementary Figure S8 and Figure S9). In patients with lower blood eosinophil levels (< 300 cells/μL) IND/GLY provided better protection against future exacerbations compared with SFC. However, in patients with higher blood eosinophil levels (≥300 cells/μL), the efficacy of IND/GLY was comparable with SFC.

### Impact of smoking status on the rates of moderate/severe exacerbations

Baseline smoking status had no impact on the rate of exacerbation (RR, 1.03 [0.92 to 1.16]; *P* = 0.603). However, in both current and ex-smokers, IND/GLY showed better efficacy in terms of reducing the rates of exacerbations compared with SFC (Supplementary Figure S10).

## Discussion

The results of this analysis demonstrated that greater baseline symptom burden, in terms of higher CAT and SGRQ scores and higher dyspnoea, is associated with higher risk of exacerbations. However, patients who had concurrent bronchitis experienced lower rates of exacerbation. Treatment differences observed between IND/GLY and SFC were in line with the overall results of the FLAME study in the majority of groups studied.

Poor symptomatic control is associated with acute exacerbations, and this is a major challenge for physicians treating COPD patients. Exacerbations severely impair health status [5] and reduce life expectancy [1]. Previous reports suggest that patients with higher health status impairment, measured using CAT and SGRQ-C, are prone to exacerbations leading to death [27–29]. Outcomes of our analysis complement previous findings by confirming that patients with higher health status impairment, demonstrated by higher baseline SGRQ and CAT scores, had higher subsequent exacerbation frequency regardless of treatment received. Dyspnoea perception may also be an important factor in reporting of exacerbations and influencing exacerbation treatment [30, 31]. Prior studies have shown that increasing dyspnoea levels, as measured using the mMRC scale, are associated with increased risk of hospital readmission and mortality [32]; therefore, baseline mMRC score can be a good predictor of mortality [33].

Our findings expand on prior knowledge on dyspnoea and its impact on exacerbation risk, through interrogation of detailed eDiary symptom data. The detailed exacerbation symptom characteristics captured by the eDiary enabled us to show that patients with lower baseline dyspnoea levels experienced exacerbations of reduced symptom severity and duration compared with patients with higher levels of dyspnoea. Therefore, our results reinforce that patient-reported dyspnoea levels are a predictor of subsequent exacerbation frequency. It should be acknowledged however that in the FLAME trial patients were included with mMRC≥2. Therefore our findings may not be generalisable to patients with lower dyspnoea burden.

Bronchitis is common in COPD, and is associated with increased risk of exacerbation and hospitalisation [14]. In the present analysis, it was surprising that patients without bronchitis had increased exacerbation frequency compared with patients with bronchitis. No significant differences were seen in exacerbation symptom severity between patients with bronchitis and without bronchitis. The relatively small number of patients in the non-bronchitis group may have reduced the power of this analysis and thus contributed to this anomaly. Response to the eDiary questionnaire was used for the determination of bronchitis, in contrast to the classical definition of chronic bronchitis consisting of chronic cough and sputum production for 3 months a year for 2 consecutive years [34]. Furthermore, the vast majority of patients (76%) were classified as having bronchitis, most likely due to the FLAME inclusion criteria resulting in enrichment with patients with a history of exacerbations. Given these factors, this result must be treated with caution. Nonetheless, in keeping with the main FLAME results, patients with bronchitis symptoms treated with IND/GLY had lower exacerbation frequency compared with those receiving SFC.

Unlike previous reports, our results did not show any impact of blood eosinophil on the future risks of exacerbations. This may be due to the smaller number of patients in higher blood eosinophil group, as well as the potential effect of the two treatment arms [21, 22, 35]. We did not observe any association between smoking status (current and ex-smokers) and the efficacy of the two treatments on the risk of exacerbations [20, 35]. Despite the evidence supporting reduced steroid responsiveness in current smokers with asthma, the efficacy of ICS in current smokers with COPD is still rather controversial. Our data suggest that a long-acting β_2_-agonist/ long-acting muscarinic antagonist is superior to a long-acting β_2_-agonist/inhaled corticosteroid combination on exacerbation prevention irrespective of smoking status in COPD and these findings warrant further analyses that may support treatment decisions.

Results of our analysis also support the superiority of IND/GLY compared with SFC observed in the FLAME study by confirming that patients receiving IND/GLY had lower exacerbation frequency than those treated with SFC in both lower and higher health status impairment groups measured by SGRQ-C scores. Bronchodilators are associated with reduced exacerbation frequency, likely in part, due to improvements in symptom tolerance [36] and this work reinforces that reduction of hyperinflation and improvement of breathlessness are key goals in reducing the burden (both frequency and symptomatic intensity) of COPD exacerbations. Indeed, aside from lower baseline dyspnoea being associated with milder exacerbation severity, no relationship was seen between baseline individual symptom severity and exacerbation symptom burden. This was not the case for the group of patients without bronchitis or patients with high blood eosinophil levels, but this may well be related to the lack of power due to the very small size of this group. In both current and ex-smokers, IND/GLY showed better protection from exacerbation compared with SFC. Therefore, whilst the present analysis has reaffirmed both the strong relationship between disease severity and exacerbation frequency, and the importance of dyspnoea in COPD patients, it appears that the intensity of other stable state symptoms is not a key driver of exacerbation symptom severity. This may be because exacerbation severity is largely driven by exacerbation triggers such as respiratory viruses and bacteria [37].

As is inherent in all post hoc analyses, the present analysis has certain limitations. The group sizes are smaller (e.g. the number of patients with bronchitis was markedly larger than the patients without bronchitis) and, hence, all between-treatment results of this analysis should be interpreted with caution. However, the careful daily reporting of symptoms during the run-in period via an eDiary increases the reliability of symptom evaluation. Moreover, these analyses were performed on clinical trial data from a controlled setting, so there are advantages in data collection that eliminates the problem of missing data versus previous studies.

## Conclusions

COPD patients with higher levels of dyspnoea at baseline as well as those with greater impairment in health status experience increased exacerbation frequency. Our results suggest that future studies on novel exacerbation therapies should consider targeting patients with higher symptom burden at baseline in addition to a history of previous exacerbations.

## Supplementary information

**Additional file 1.**

## Data Availability

Novartis is committed to sharing with qualified external researchers, access to patient-level data and supporting clinical documents from eligible studies. These requests are reviewed and approved by an independent review panel on the basis of scientific merit. All data provided is anonymized to respect the privacy of patients who have participated in the trial in line with applicable laws and regulations. This trial data availability is according to the criteria and process described on www.clinicalstudydatarequest.com

## Abbreviations

COPD: Chronic obstructive pulmonary disease
CAT: COPD Assessment Test
eDiary: electronic diary
ESCO: Exacerbations and Symptoms in COPD
FEV_1_: Forced expiratory volume in 1 s
GOLD: Global initiative for chronic obstructive lung disease
IND/GLY: Indacaterol/glycopyrronium
mMRC: modified Medical Research Council
SFC: Salmeterol/fluticasone
SGRQ-C: St. George’s Respiratory Questionnaire for COPD

## References

[1] Vogelmeier CF, Criner GJ, Martinez FJ, Anzueto A, Barnes PJ, Bourbeau J, Celli BR, Chen R, Decramer M, Fabbri LM (2017). Global strategy for the diagnosis, management, and prevention of chronic obstructive lung disease 2017 report. GOLD executive summary. Am J Respir Crit Care Med.

[2] Jones PW (2005). St. George's respiratory questionnaire: MCID. COPD..

[3] Kocks JW, Tuinenga MG, Uil SM, van den Berg JW, Stahl E, van der Molen T (2006). Health status measurement in COPD: the minimal clinically important difference of the clinical COPD questionnaire. Respir Res.

[4] Mackay AJ, Donaldson GC, Patel AR, Jones PW, Hurst JR, Wedzicha JA (2012). Usefulness of the chronic obstructive pulmonary disease assessment test to evaluate severity of COPD exacerbations. Am J Respir Crit Care Med.

[5] Seemungal TA, Donaldson GC, Paul EA, Bestall JC, Jeffries DJ, Wedzicha JA (1998). Effect of exacerbation on quality of life in patients with chronic obstructive pulmonary disease. Am J Respir Crit Care Med.

[6] Martinez FJ, Fabbri LM, Ferguson GT, Orevillo C, Darken P, Martin UJ, Reisner C (2017). Baseline symptom score impact on benefits of Glycopyrrolate/Formoterol metered dose inhaler in COPD. Chest..

[7] Casanova C, Marin JM, Martinez-Gonzalez C, de Lucas-Ramos P, Mir-Viladrich I, Cosio B, Peces-Barba G, Solanes-Garcia I, Aguero R, Feu-Collado N (2015). Differential effect of modified Medical Research Council dyspnea, COPD assessment test, and clinical COPD questionnaire for symptoms evaluation within the new GOLD staging and mortality in COPD. Chest..

[8] de Oliveira JC, de Carvalho AI, de Oliveira Beloto AC, Santos IR, Filho FS, Sampaio LM, Donner CF, Oliveira LV (2013). Clinical significance in COPD patients followed in a real practice. Multidiscip Respir Med.

[9] Mullerova H, Lu C, Li H, Tabberer M (2014). Prevalence and burden of breathlessness in patients with chronic obstructive pulmonary disease managed in primary care. PLoS One.

[10] Punekar YS, Shukla A, Mullerova H (2014). COPD management costs according to the frequency of COPD exacerbations in UK primary care. Int J Chron Obstruct Pulmon Dis.

[11] Lahousse L, Seys LJM, Joos GF, Franco OH, Stricker BH, Brusselle GG. Epidemiology and impact of chronic bronchitis in chronic obstructive pulmonary disease. Eur Respir J. 2017;50. 10.1183/13993003.02470-2016 PMC5593375 28798087

[12] Tsiligianni I, Mezzi K, Fucile S, Kostikas K, Shen S, Banerji D, Fogel R (2017). Response to Indacaterol/Glycopyrronium (IND/GLY) by sex in patients with COPD: a pooled analysis from the IGNITE program. COPD.

[13] Allinson JP, Hardy R, Donaldson GC, Shaheen SO, Kuh D, Wedzicha JA (2016). The presence of chronic mucus Hypersecretion across adult life in relation to chronic obstructive pulmonary disease development. Am J Respir Crit Care Med.

[14] Kim V, Criner GJ (2015). The chronic bronchitis phenotype in chronic obstructive pulmonary disease: features and implications. Curr Opin Pulm Med.

[15] Vestbo J, Prescott E, Lange P (1996). Association of chronic mucus hypersecretion with FEV1 decline and chronic obstructive pulmonary disease morbidity. Copenhagen City heart study group. Am J Respir Crit Care Med.

[16] Kostikas K, Aalamian-Mattheis M, Pagano VA, Nunez X, Fogel R, Patalano F, Clemens A (2018). Early changes in eDiary COPD symptoms predict clinically relevant treatment response at 12 weeks: analysis from the CRYSTAL study. COPD.

[17] Kulich K, Keininger DL, Tiplady B, Banerji D (2015). Symptoms and impact of COPD assessed by an electronic diary in patients with moderate-to-severe COPD: psychometric results from the SHINE study. Int J Chron Obstruct Pulmon Dis.

[18] Yun JH, Lamb A, Chase R, Singh D, Parker MM, Saferali A, Vestbo J, Tal-Singer R, Castaldi PJ, Silverman EK, Hersh CP (2018). Blood eosinophil count thresholds and exacerbations in patients with chronic obstructive pulmonary disease. J Allergy Clin Immunol.

[19] Schumann DM, Tamm M, Kostikas K, Stolz D (2019). Stability of the blood Eosinophilic phenotype in stable and exacerbated COPD. Chest.

[20] Josephs L, Culliford D, Johnson M, Thomas M (2017). Improved outcomes in ex-smokers with COPD: a UK primary care observational cohort study. Eur Respir J.

[21] Lipson DA, Barnhart F, Brealey N, Brooks J, Criner GJ, Day NC, Dransfield MT, Halpin DMG, Han MK, Jones CE (2018). Once-daily single-inhaler triple versus dual therapy in patients with COPD. N Engl J Med.

[22] Papi A, Vestbo J, Fabbri L, Corradi M, Prunier H, Cohuet G, Guasconi A, Montagna I, Vezzoli S, Petruzzelli S (2018). Extrafine inhaled triple therapy versus dual bronchodilator therapy in chronic obstructive pulmonary disease (TRIBUTE): a double-blind, parallel group, randomised controlled trial. Lancet.

[23] Wedzicha JA, Banerji D, Chapman KR, Vestbo J, Roche N, Ayers RT, Thach C, Fogel R, Patalano F, Vogelmeier CF (2016). Indacaterol-Glycopyrronium versus Salmeterol-fluticasone for COPD. N Engl J Med.

[24] Global Strategy for the Diagnosis, Management and Prevention of COPD, Global Initiative for Chronic Obstructive Lung Disease (GOLD). Available at: www.goldcopd.org. Accessed on 6 Dec 2019.

[25] Anthonisen NR, Manfreda J, Warren CP, Hershfield ES, Harding GK, Nelson NA (1987). Antibiotic therapy in exacerbations of chronic obstructive pulmonary disease. Ann Intern Med.

[26] Mackay AJ, Kostikas K, Murray L, Martinez FJ, Miravitlles M, Donaldson G, Banerji D, Patalano F, Wedzicha JA (2018). Patient-reported outcomes for the detection, quantification, and evaluation of chronic obstructive pulmonary disease exacerbations. Am J Respir Crit Care Med.

[27] Lee SD, Huang MS, Kang J, Lin CH, Park MJ, Oh YM, Kwon N, Jones PW, Sajkov D (2014). The COPD assessment test (CAT) assists prediction of COPD exacerbations in high-risk patients. Respir Med.

[28] Mullerova H, Gelhorn H, Wilson H, Benson VS, Karlsson N, Menjoge S, Rennard SI, Tabberer M, Tal-Singer R, Merrill D, Jones PW (2017). St George's respiratory questionnaire score predicts outcomes in patients with COPD: analysis of individual patient data in the COPD biomarkers qualification consortium database. Chronic Obstr Pulm Dis.

[29] Sajkov D, Huang M-S, Kang J, Lin C-H, Park MJ, Kwon N, Lee S-D (2012). The predictive value of the COPD assessment test (CAT) for acute exacerbations in patients with chronic obstructive pulmonary disease (COPD). Eur Respir J.

[30] Scioscia G, Blanco I, Arismendi E, Burgos F, Gistau C, Foschino Barbaro MP, Celli B, O'Donnell DE, Agusti A (2017). Different dyspnoea perception in COPD patients with frequent and infrequent exacerbations. Thorax.

[31] Vijayasaratha K, Stockley RA (2008). Reported and unreported exacerbations of COPD: analysis by diary cards. Chest..

[32] Steer J, Norman EM, Afolabi OA, Gibson GJ, Bourke SC (2012). Dyspnoea severity and pneumonia as predictors of in-hospital mortality and early readmission in acute exacerbations of COPD. Thorax..

[33] Nishimura K, Izumi T, Tsukino M, Oga T (2002). Dyspnea is a better predictor of 5-year survival than airway obstruction in patients with COPD. Chest..

[34] Fletcher CM, Elmes PC, Fairbairn AS, Wood CH (1959). The significance of respiratory symptoms and the diagnosis of chronic bronchitis in a working population. Br Med J.

[35] Vogelmeier CF, Kostikas K, Fang J, Tian H, Jones B, Morgan CL, Fogel R, Gutzwiller FS, Cao H (2019). Evaluation of exacerbations and blood eosinophils in UK and US COPD populations. Respir Res.

[36] Beeh KM, Burgel PR, Franssen FME, Lopez-Campos JL, Loukides S, Hurst JR, Flezar M, Ulrik CS, Di Marco F, Stolz D (2017). How do dual long-acting bronchodilators prevent exacerbations of chronic obstructive pulmonary disease?. Am J Respir Crit Care Med.

[37] Welte T, Miravitlles M (2014). Viral, bacterial or both? Regardless, we need to treat infection in COPD. Eur Respir J.

