# Supplementary material for: Impact of baseline symptoms and health status on COPD exacerbations in the FLAME study
Source: Respir Res. 2020 Apr 22;21:93. doi: 10.1186/s12931-020-01354-8 (PMC7179005; doi:10.1186/s12931-020-01354-8)
Supplement: Supplementary file 1 — Additional file 1. [file 12931_2020_1354_MOESM1_ESM.docx]

## **Impact of baseline symptoms and health status on COPD exacerbations in the FLAME study**

## Alexander J. Mackay^1*^, Konstantinos Kostikas^2#^, Nicolas Roche^3^, Stefan-Marian Frent^4^,

## Petter Olsson^5^, Pascal Pfister^6^, Pritam Gupta^7^, Francesco Patalano^6^, Donald Banerji^8^,

## Jadwiga A. Wedzicha^1^

## ^1^National Heart and Lung Institute, Imperial College London, London, United Kingdom; ^2^Respiratory Medicine Department, University of Ioannina Medical School, Ioannina, Greece; ^3^Pneumologie Hôpital Cochin (APHP), Université Paris Descartes (EA2511), Paris, France; ^4^Department of Pulmonology, University of Medicine and Pharmacy Timisoara, Timisoara, Romania; ^5^Novartis Sverige AB, Täby, Sweden; ^6^Novartis Pharma AG, Basel, Switzerland; ^7^Novartis Healthcare Pvt. Ltd., Hyderabad, India; ^8^Novartis Pharmaceuticals Corporation, East Hanover, NJ, United States

*At the time of the study conducted AJM was a European Respiratory Society Fellow at Novartis Pharma AG, Basel, Switzerland, and he is now working with AstraZeneca, Gothenburg, Sweden. ^#^KK was an employee of Novartis Pharma AG, Basel, Switzerland at the time of the conduct of this study.

# Corresponding author

Prof. Jadwiga A. Wedzicha

National Heart and Lung Institute

Imperial College London, London, United Kingdom

Tel: +442075947947; E-mail: [j.wedzicha@imperial.ac.uk](mailto:j.wedzicha@imperial.ac.uk)

## **Figure S1. Study design [1]**

| 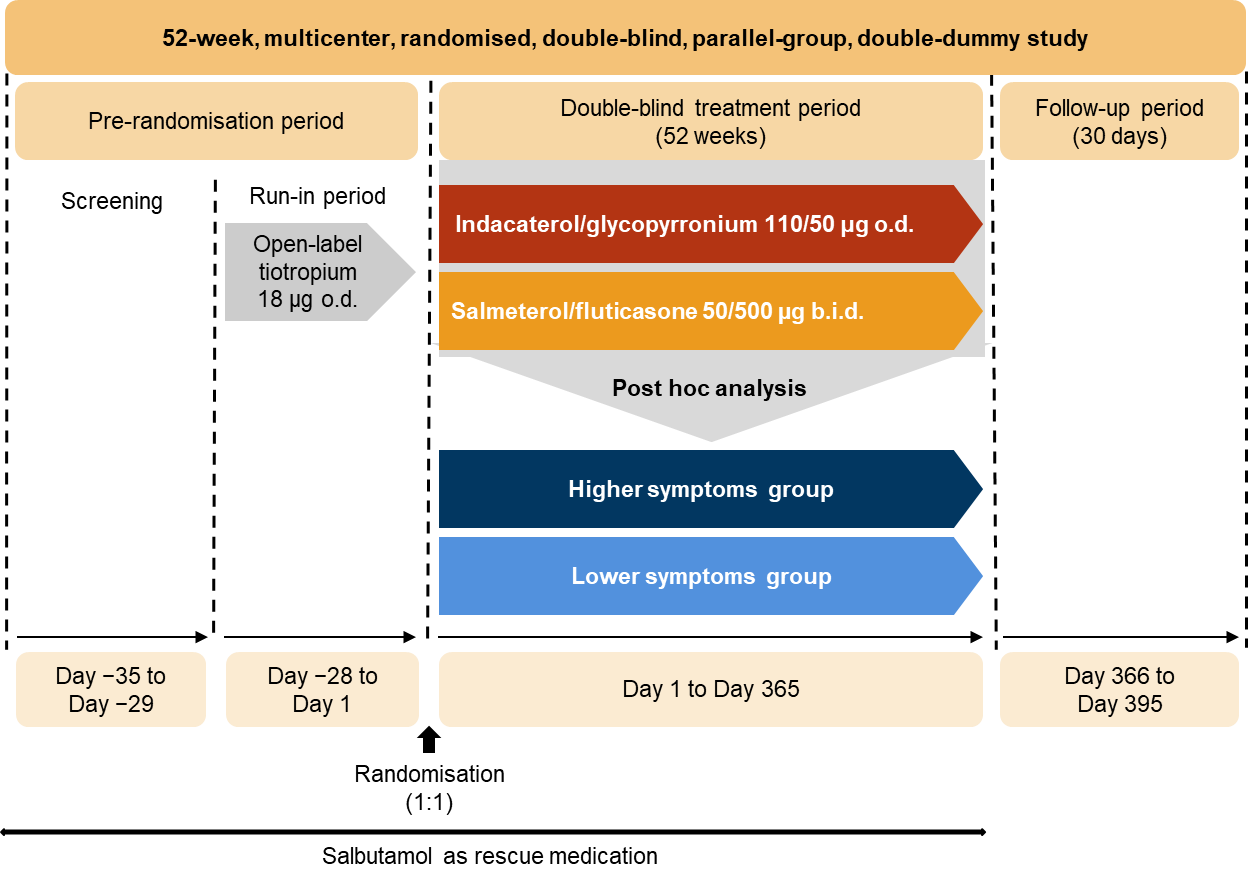 |
| --- |
| b.i.d., twice daily; o.d., once daily  Baseline symptoms were evaluated in terms of higher or lower health status impairments (measured via total scores of COPD assessment test and St. George’s Respiratory Questionnaire) and dyspnoea and bronchitis (measured via electronic diary) |

**Figure S2. Hypothetical total symptom burden (measured using the eDiary data) at the onset and during an exacerbation [2]**

| **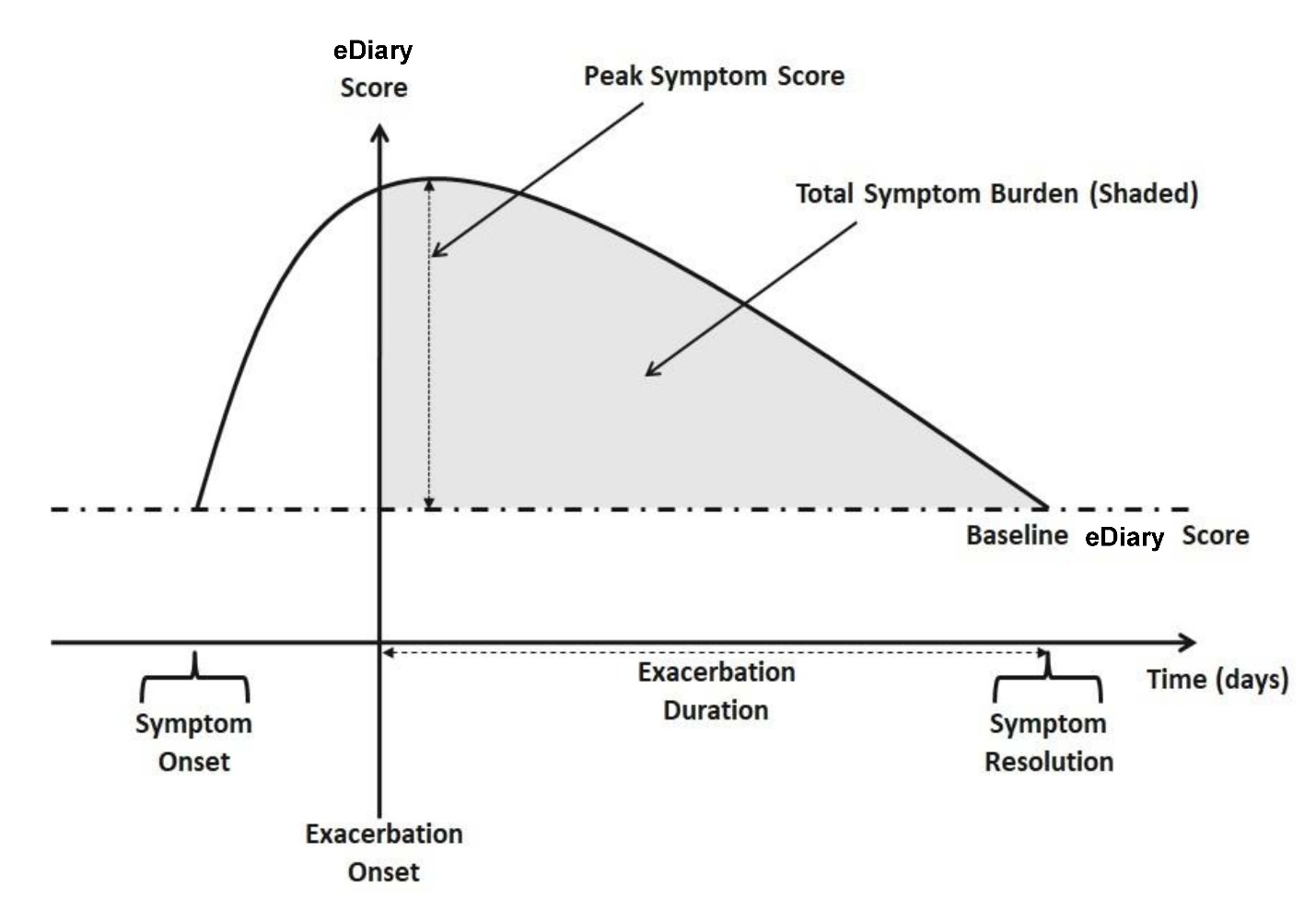** |
| --- |
| Reprinted with permission of the American Thoracic Society. Copyright © 2018 American Thoracic Society.  Mackay AJ, et al. Am J Respir Crit Care Med. 2018;198(6):730-738 [2]. The American Journal of Respiratory and Critical Care Medicine is an official journal of the American Thoracic Society.  eDiary, electronic diary |

**Figure S3a. Mean percentage of days on moderate or severe exacerbation and mean peak symptom scores (eDiary) during the period of moderate or severe exacerbations in patients with high or low CAT score**

| **Mean % of days on moderate or severe exacerbation by CAT score** |
| --- |
| **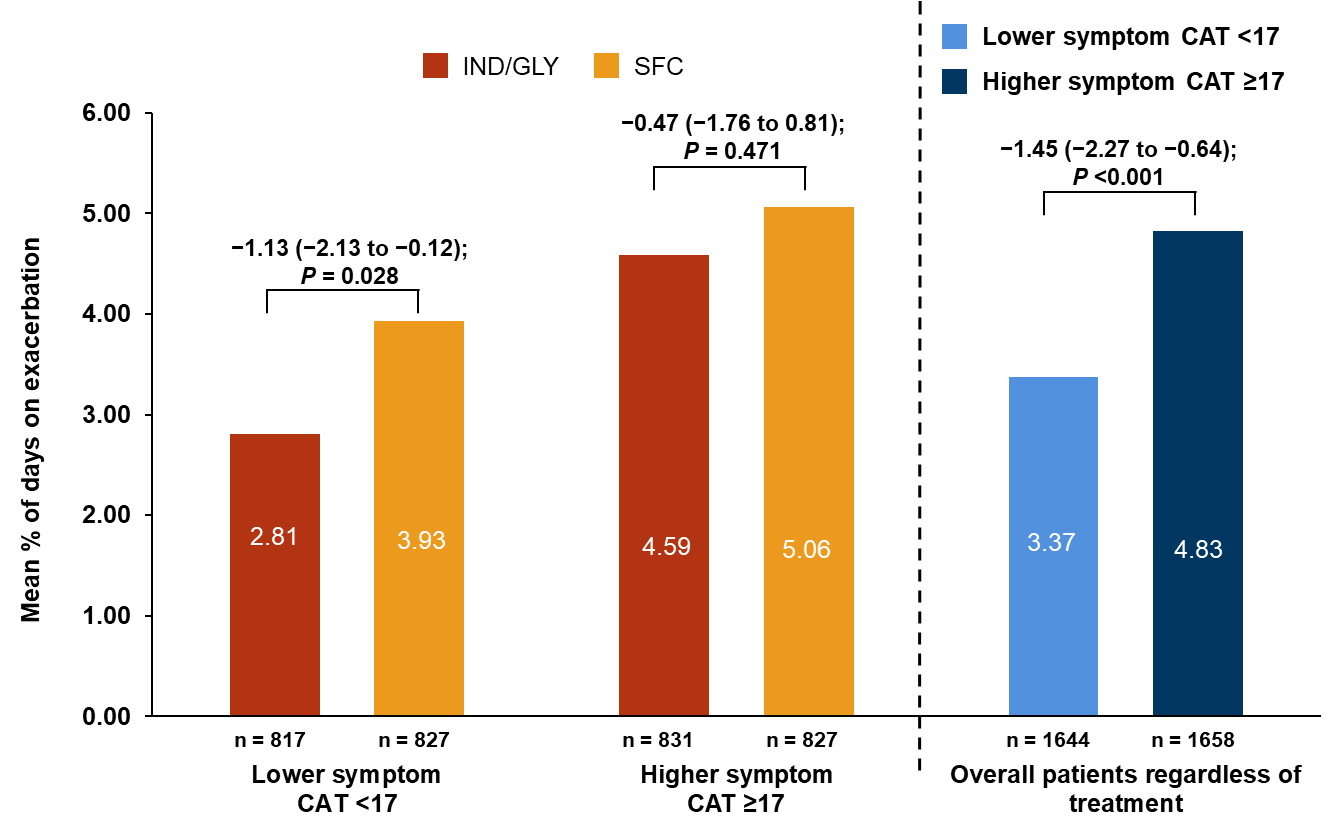** |
| **Mean peak symptom scores by CAT score** |
| **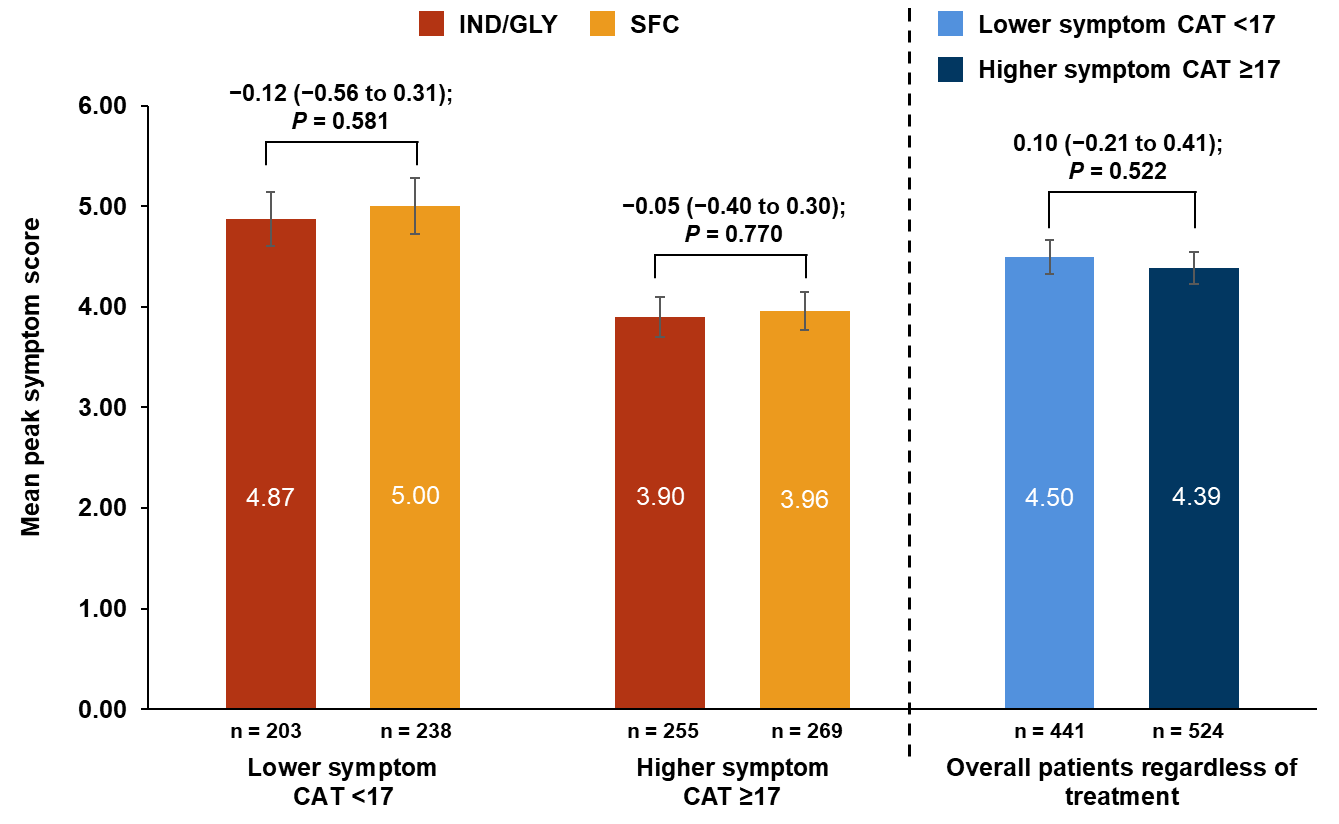** |
| n, number of patients in each group.  In the first panel of the figure data are presented as percentage mean; in the second panel of the figure data are presented as LSM ± SE. Differences between groups are presented as mean difference or LSM difference with 95% CI.  CAT was used to measure health status; patients were divided into higher (CAT score ≥17) or lower (CAT score <17) health status impairment groups with split at median based on CAT levels obtained at Day 1  CAT, COPD Assessment Test; eDiary, electronic diary; IND/GLY, indacaterol/glycopyrronium 110/50 µg once daily; LSM, least squares mean; SFC, salmeterol/fluticasone 50/500 µg twice daily |

**Figure S3b. Mean percentage of days on moderate or severe exacerbation and mean peak symptom scores (eDiary) during the period of moderate or severe exacerbations in patients with high or low SGRQ-C score**

| **Mean % of days on moderate or severe exacerbation by SGRQ-C score** |
| --- |
| **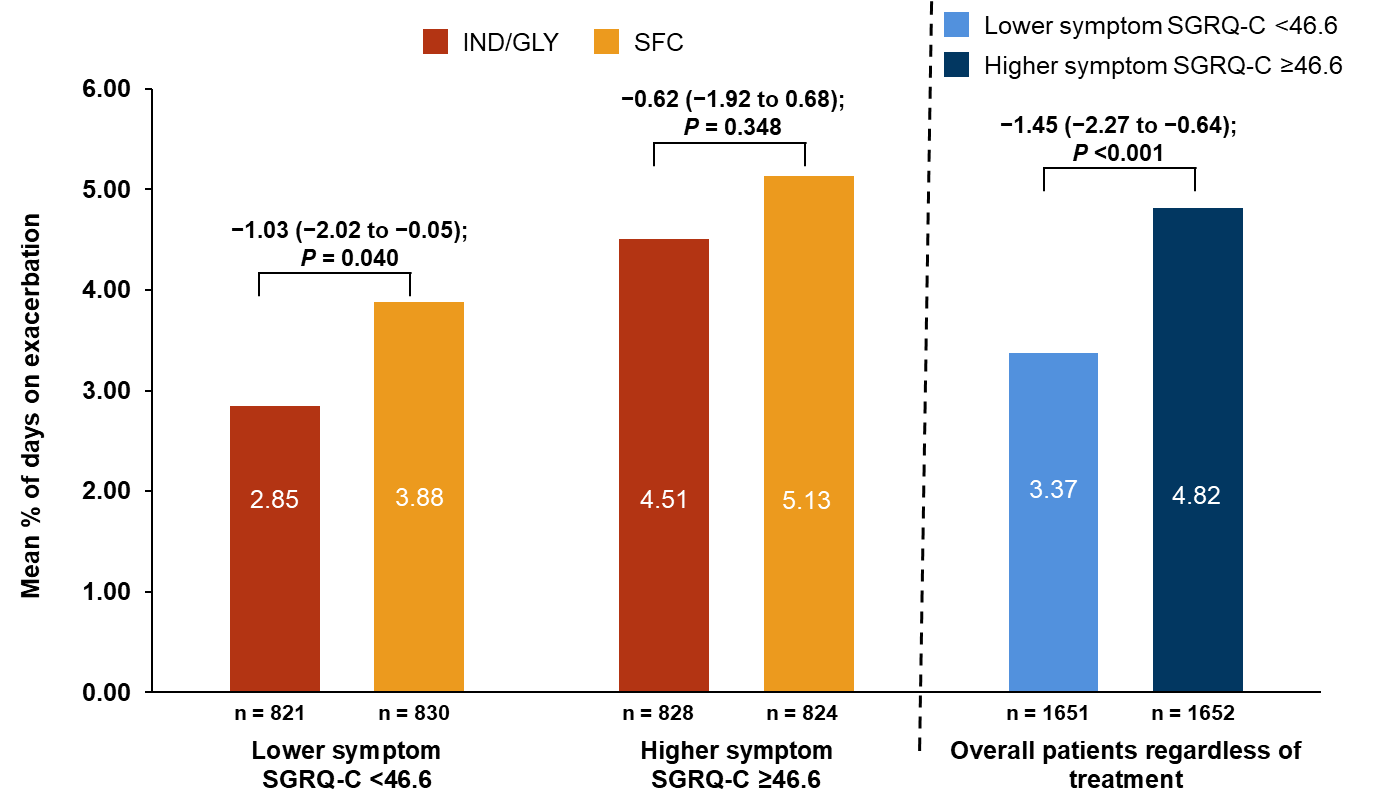** |
| **Mean peak symptom scores by SGRQ-C score** |
| **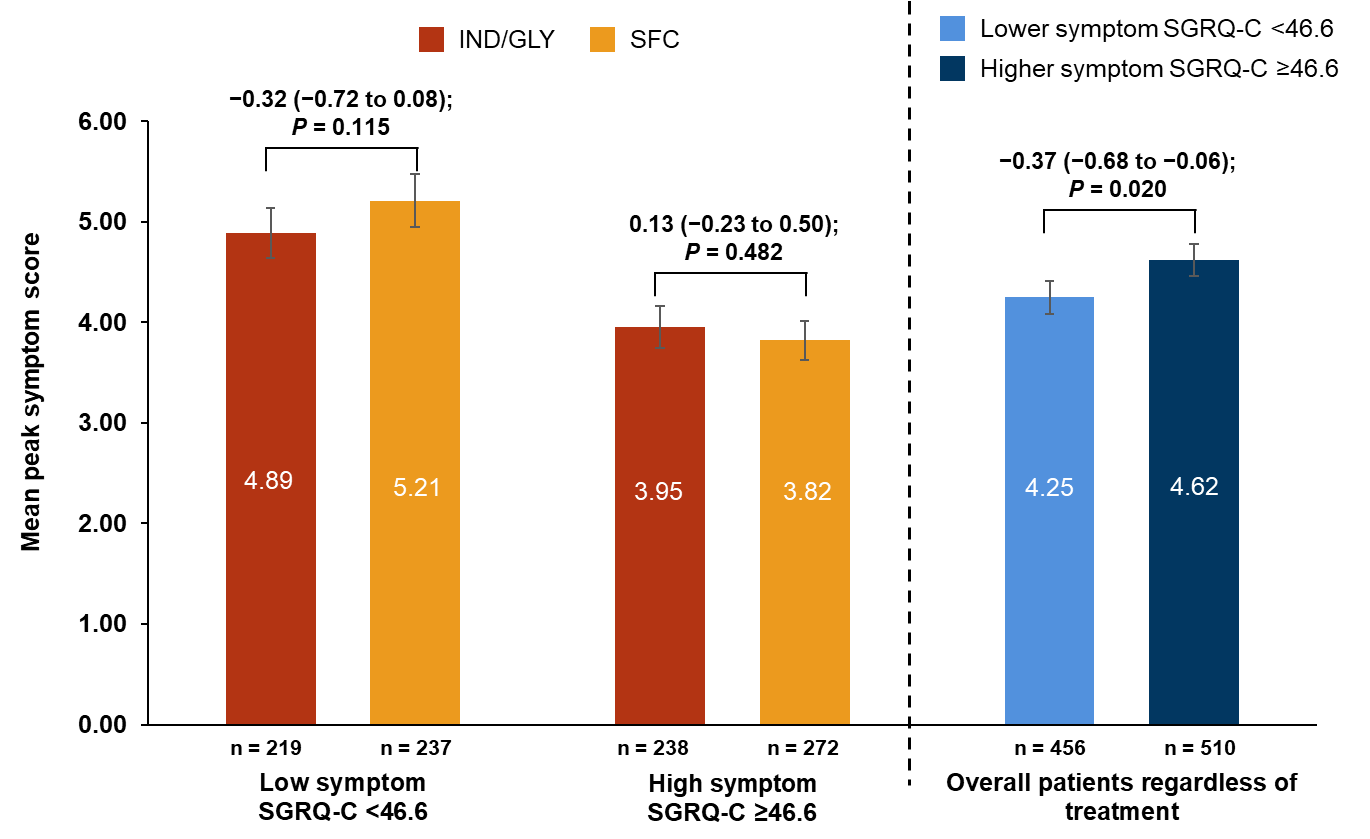** |
| n, number of patients in each group.  In the first panel of the figure data are presented as percentage mean; in the second panel of the figure data are presented as LSM ± SE. Differences between groups are presented as mean difference or LSM difference with 95% CI.  SGRQ-C was used to measure health status; patients were divided into higher (SGRQ-C score ≥46.6) or lower (SGRQ-C score <46.6) health status impairment groups with split at median based on SGRQ levels obtained at Day 1. eDiary, electronic diary; IND/GLY, indacaterol/glycopyrronium 110/50 µg once daily; LSM, least squares mean; SFC, salmeterol/fluticasone 50/500 µg twice daily; SGRQ-C, St. George’s Respiratory Questionnaire for COPD |

**Figure S4. (a) Mean percentage of days on moderate or severe exacerbation and (b) mean peak symptom scores (eDiary) during the period of moderate or severe exacerbations in patients with higher and lower dyspnoea**

| **a** | **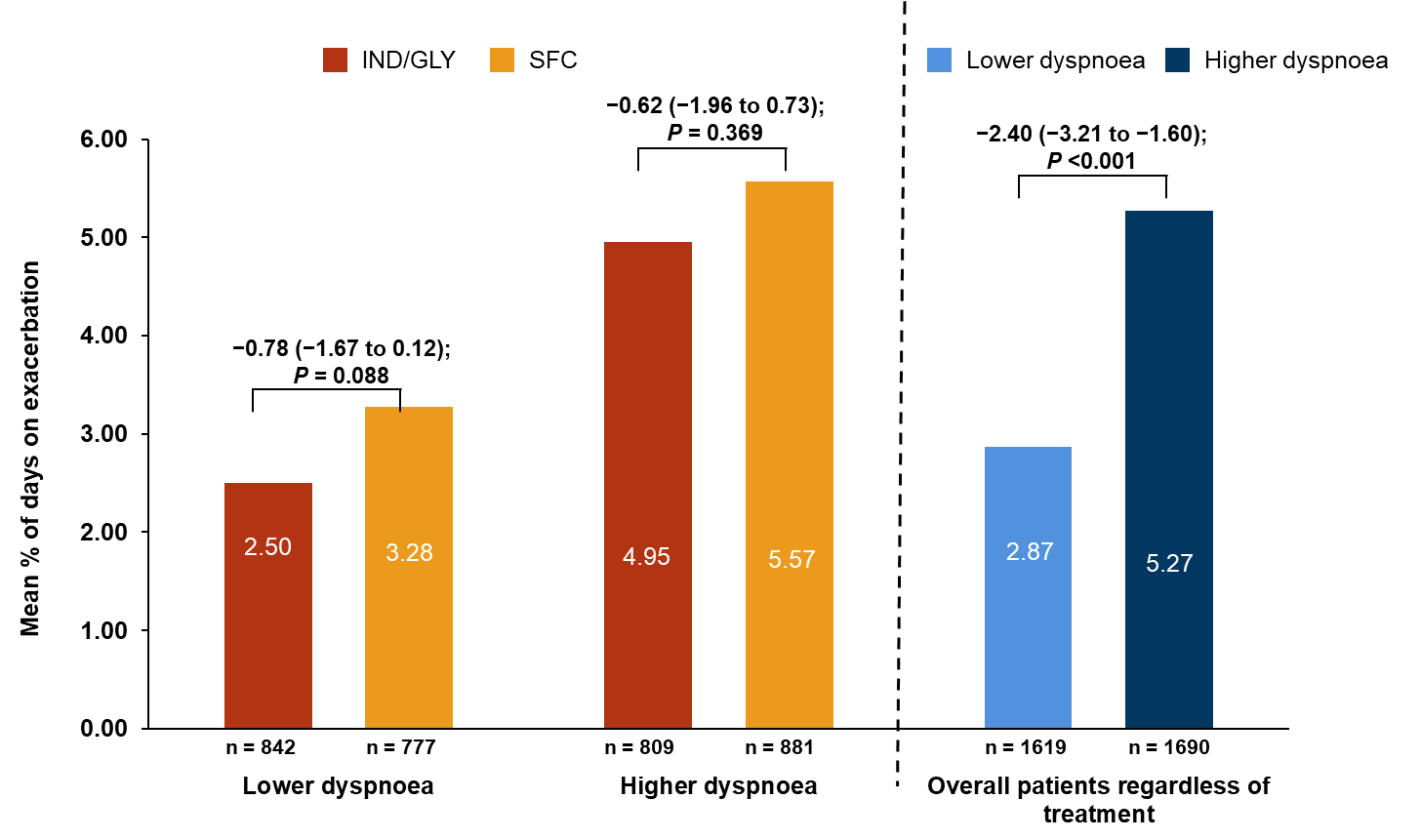** |
| --- | --- |
| **b** | **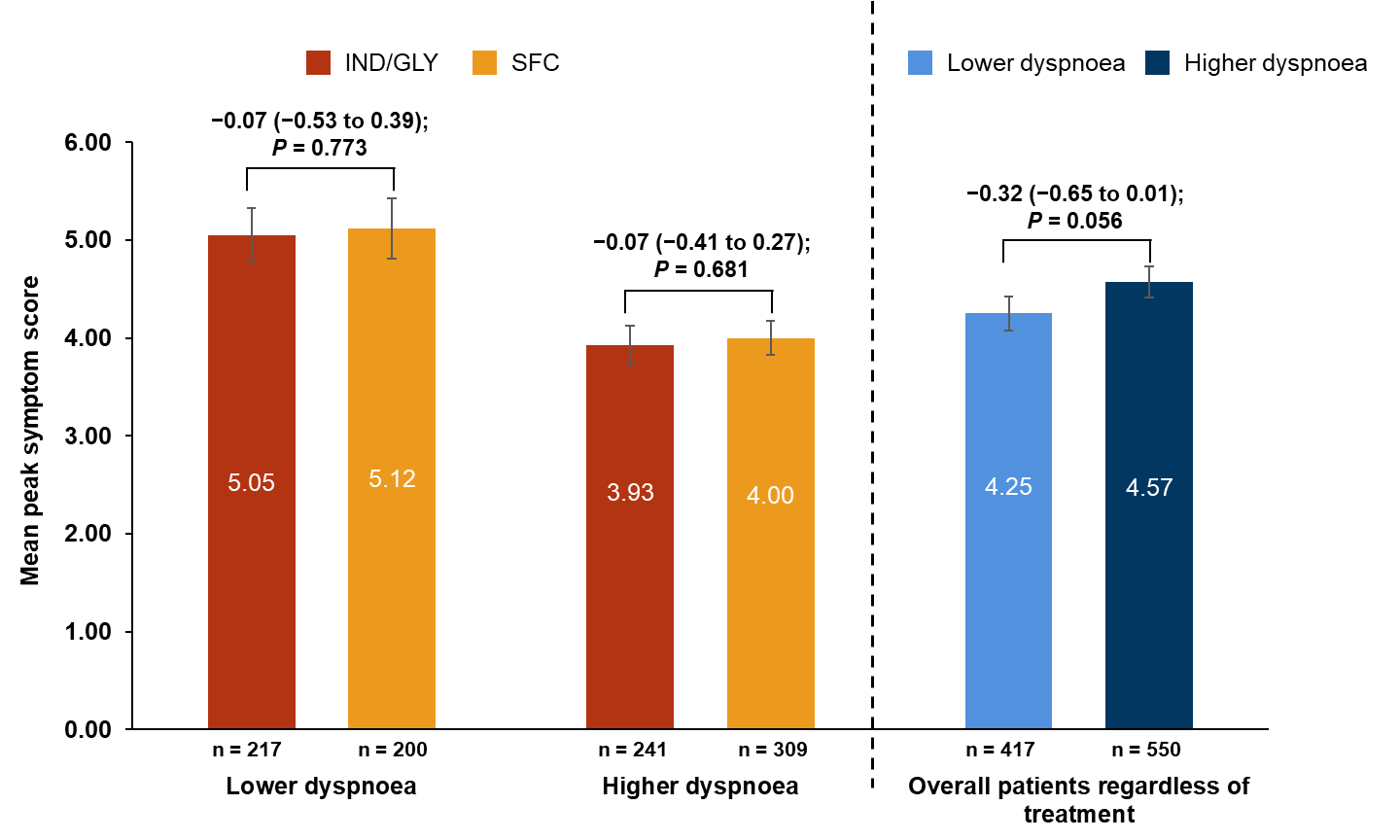** |
| n, number of patients in each group.  Patients were divided into higher or lower dyspnoea groups with split at median based on daily highest dyspnoea scores on eDiary averaged over the run-in period (approximately 28 days) including the morning assessments at Day 1  In figure S3a data are presented as percentage mean; in figure S3b data are presented as LSM ± SE.  Differences between groups are presented as mean difference or LSM difference with 95% CI.  eDiary, electronic diary; IND/GLY, indacaterol/glycopyrronium 110/50 µg once daily; LSM, least squares mean; SFC, salmeterol/fluticasone 50/500 µg twice daily | |

**Figure S5. (a) Mean percentage of days on moderate or severe exacerbation and (b) mean peak symptom scores (eDiary) during the period of moderate or severe exacerbations in patients with or without bronchitis**

| **a** | **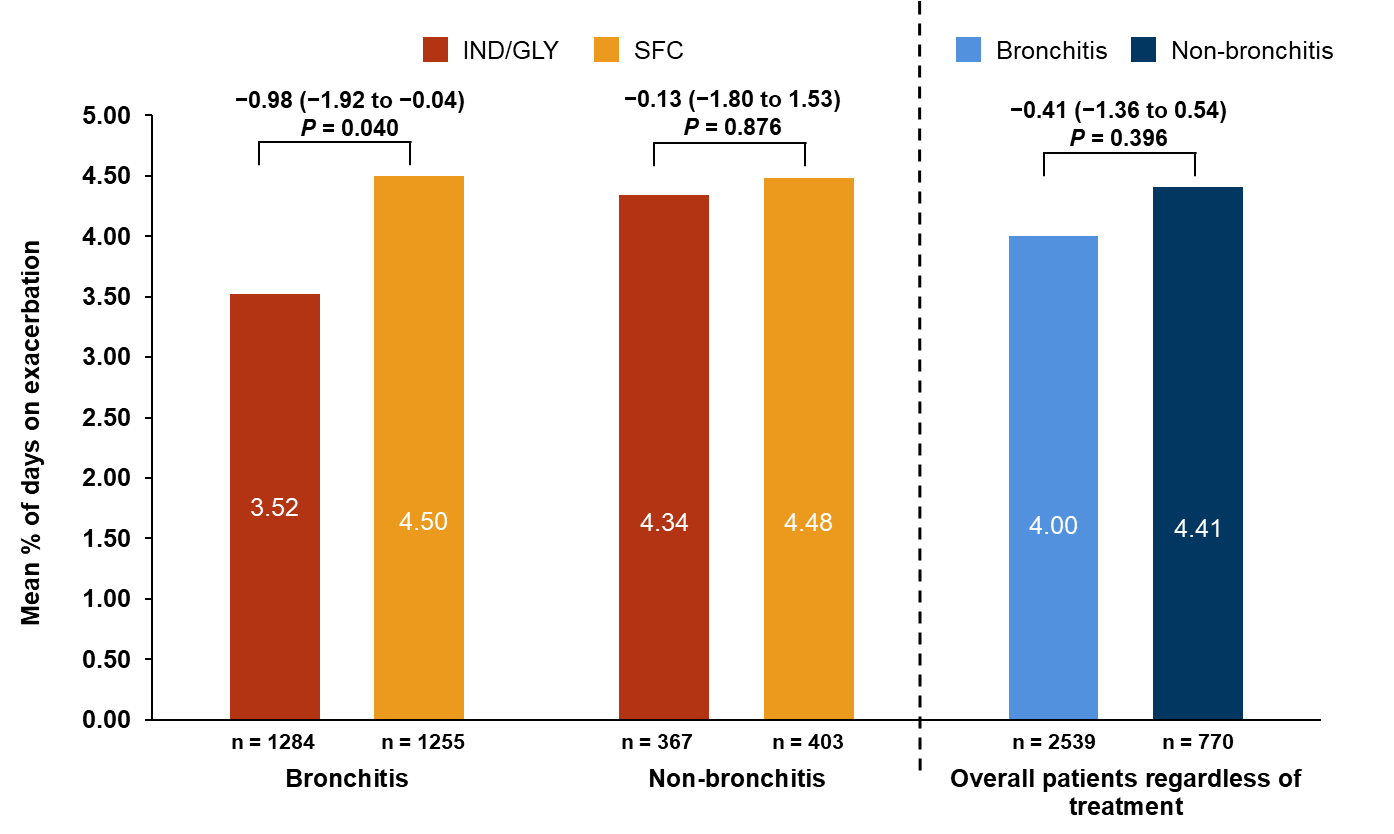** |
| --- | --- |
| **b** | **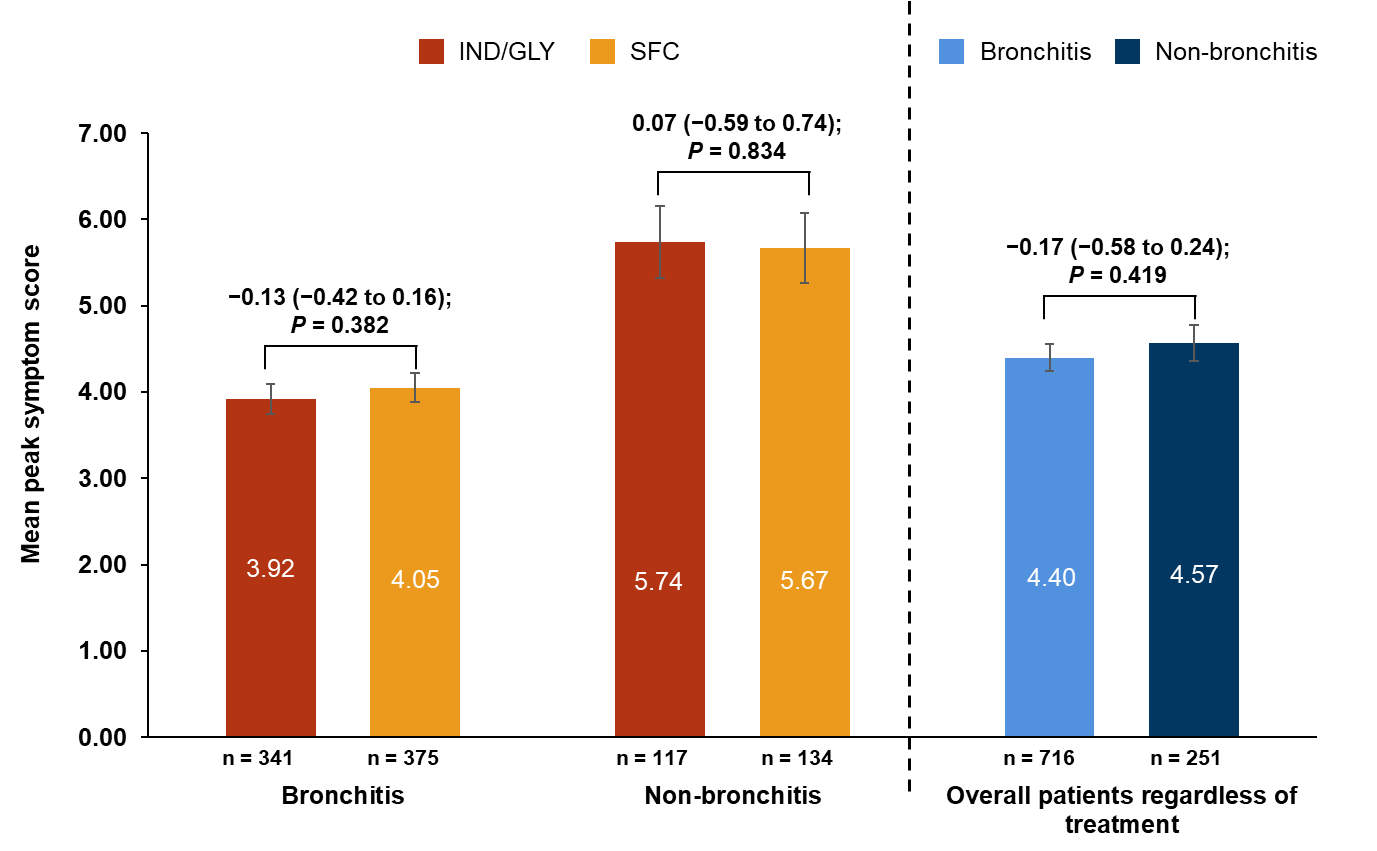** |
| n, number of patients in each group.  In figure S4a data are presented as percentage mean; in figure S4b data are presented as LSM ± SE. Differences between groups are presented as mean difference or LSM difference with 95% CI.  Bronchitis was evaluated based on patients’ response to a specific question in the Exacerbations and Symptoms in COPD (ESCO) eDiary: “How much sputum did you produce in the past 12 hours?” on a scale of 0 to 3 (0 = none; 1 = <5 mL/1 teaspoon [tsp]; 2 = 5–25 mL/1–5 tsp; 3 = >25 mL/5 tsp). Bronchitis and non-bronchitis patients were defined as those with daily highest sputum volume score of ≥1 (bronchitis) or <1 (non-bronchitis), respectively, for ≥50% of the time during the run-in period including Day 1.  eDiary, electronic diary; IND/GLY, indacaterol/glycopyrronium 110/50 µg once daily; LSM, least squares mean; SFC, salmeterol/fluticasone 50/500 µg twice daily | |

**Impact of other baseline symptoms on the rates and characteristics of moderate/severe exacerbations**

Patients were divided into low (eDiary score <6.5) or high (eDiary score ≥6.5) symptom burden based on median split of total electronic diary (eDiary) symptom scores (except rescue medication) averaged over the run-in, which is usually a 28-day period, including the morning assessments at Day 1. The total symptom score is based on the sum of the highest daily score each day for all the symptoms assessed via e-Diary which was to be completed twice daily.

**Figure S6. Annualised rate of moderate or severe exacerbations by baseline symptom burden measured using eDiary total scores**

| 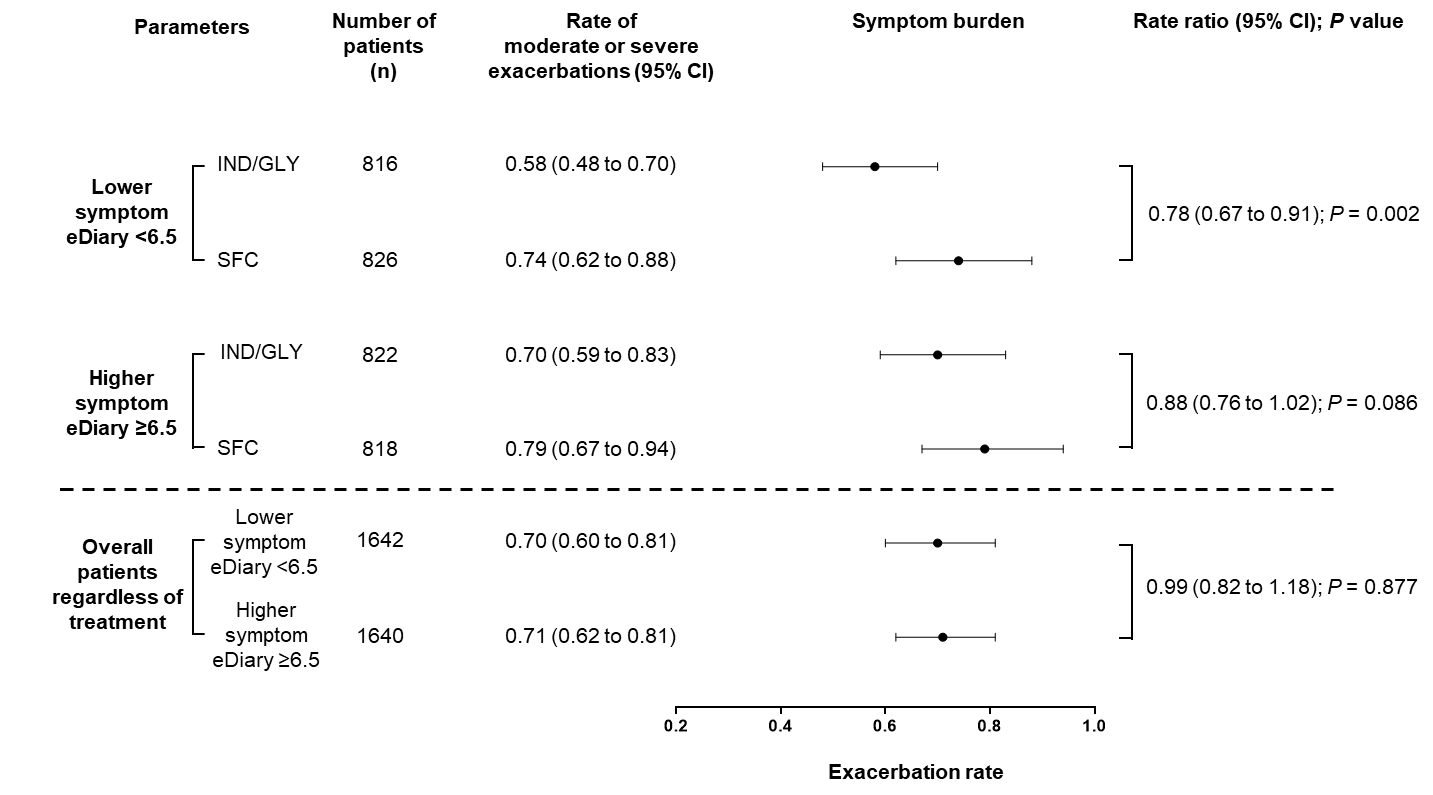 |
| --- |
| n, number of patients assessed in this analysis.  eDiary, electronic diary; IND/GLY, indacaterol/glycopyrronium 110/50 µg once daily; SFC, salmeterol/fluticasone 50/500 µg twice daily |

**Figure S7. (a) Mean percentage of days on moderate or severe exacerbation and (b) mean peak symptom scores (eDiary) during the period of moderate or severe exacerbations in patients with higher and lower symptoms**

| **a** | **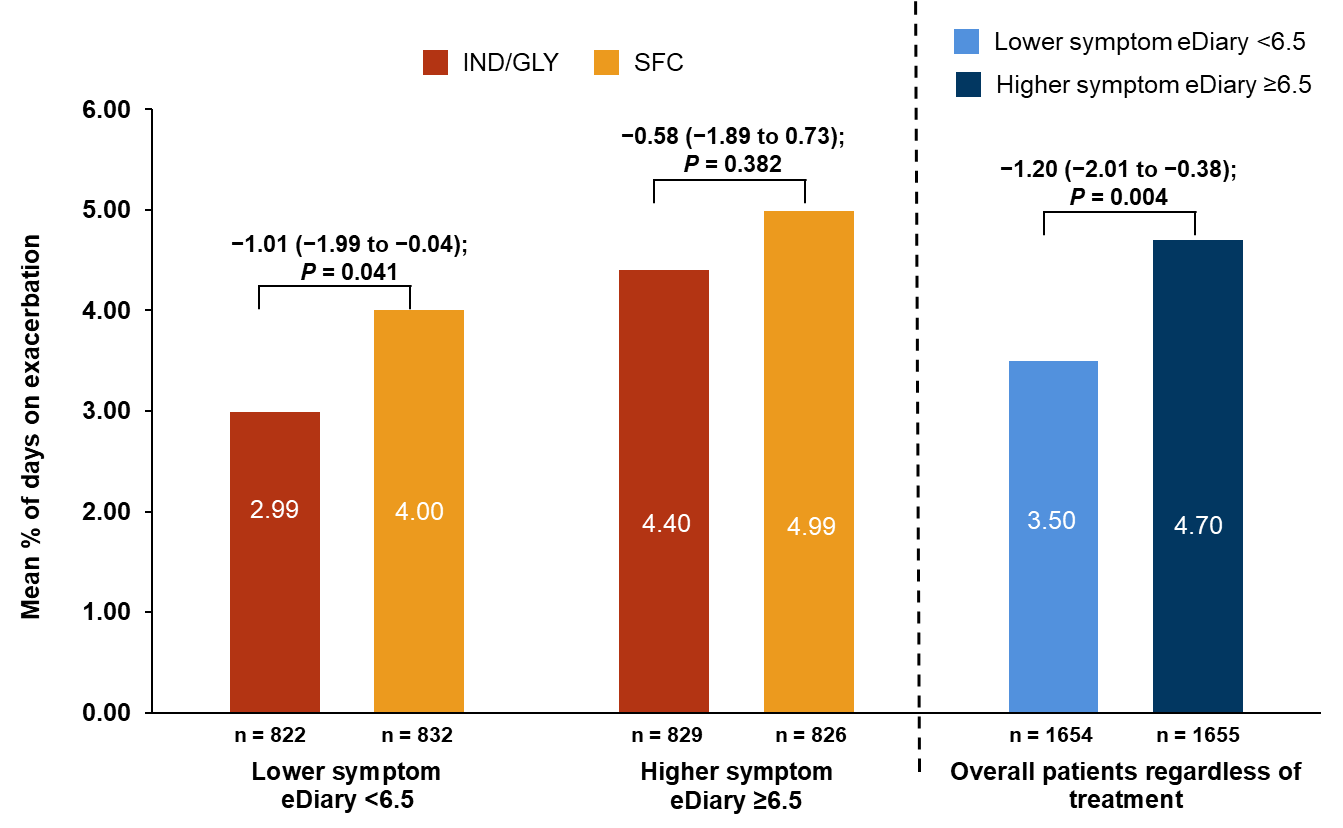** |
| --- | --- |
| **b** | **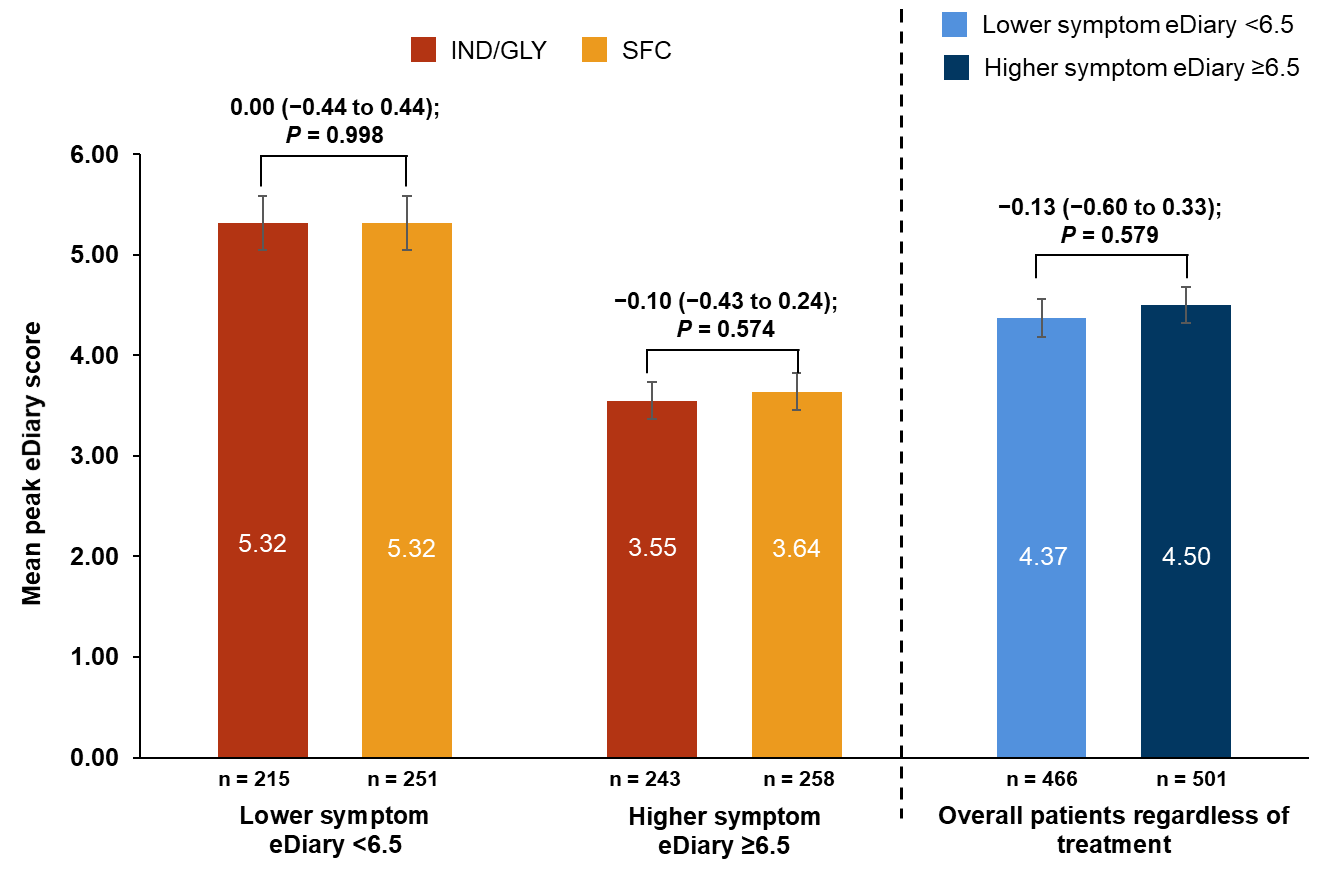** |
| n, number of patients in each group.  In figure S6a data are presented as percentage mean; in figure S6b data are presented as LSM ± SE. Differences between groups are presented as mean difference or LSM difference with 95% CI.  IND/GLY, indacaterol/glycopyrronium 110/50 µg once daily; LSM, least squares mean; SFC, salmeterol/fluticasone 50/500 µg twice daily | |

**Figure S8. Annualised rate of moderate or severe exacerbations by baseline blood eosinophil levels**

| 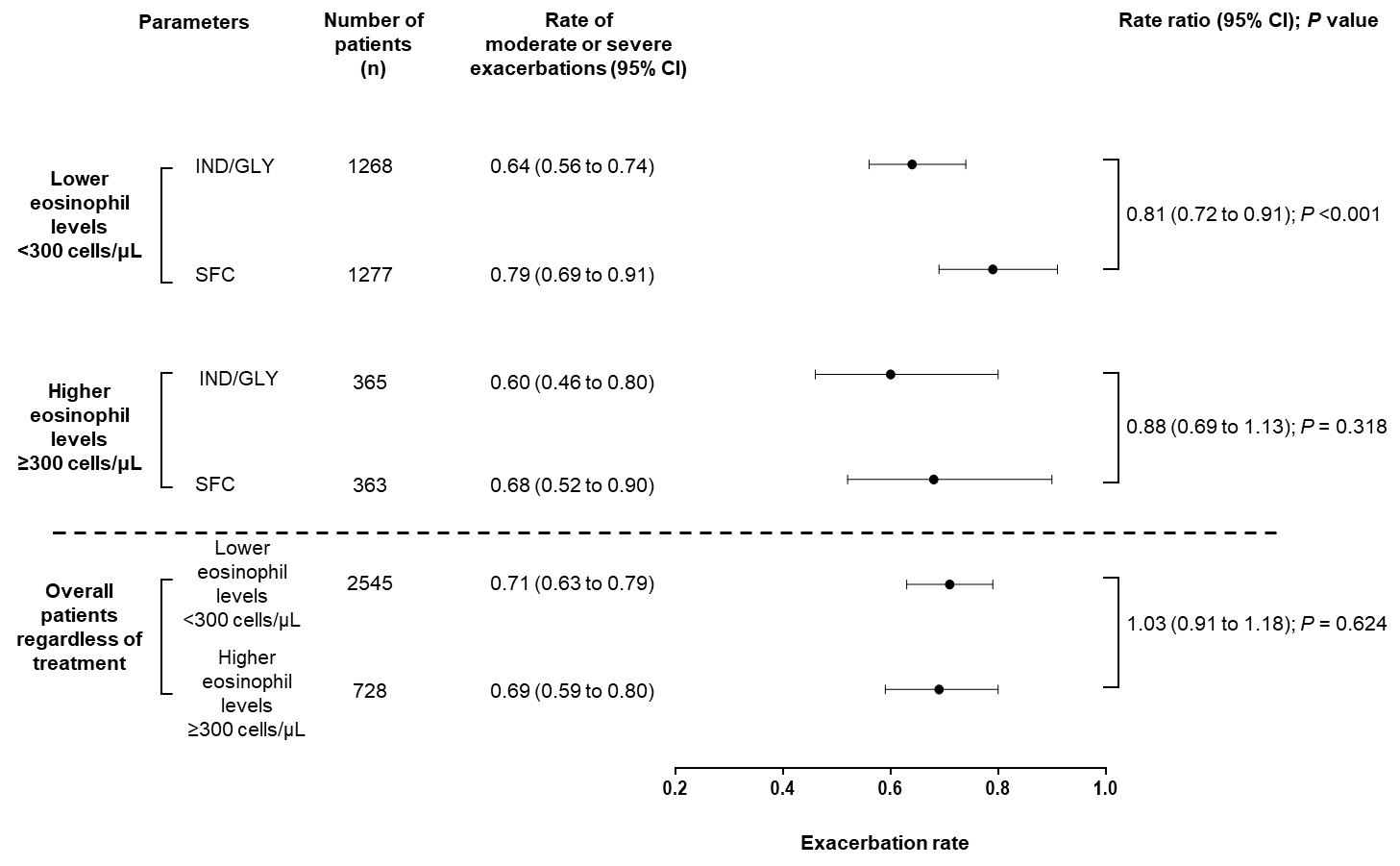 |
| --- |
| n, number of patients assessed in this analysis.  eDiary, electronic diary; IND/GLY, indacaterol/glycopyrronium 110/50 µg once daily; SFC, salmeterol/fluticasone 50/500 µg twice daily |

**Figure S9. Mean percentage of days on moderate or severe exacerbation in patients with higher and lower blood eosinophil levels**

| **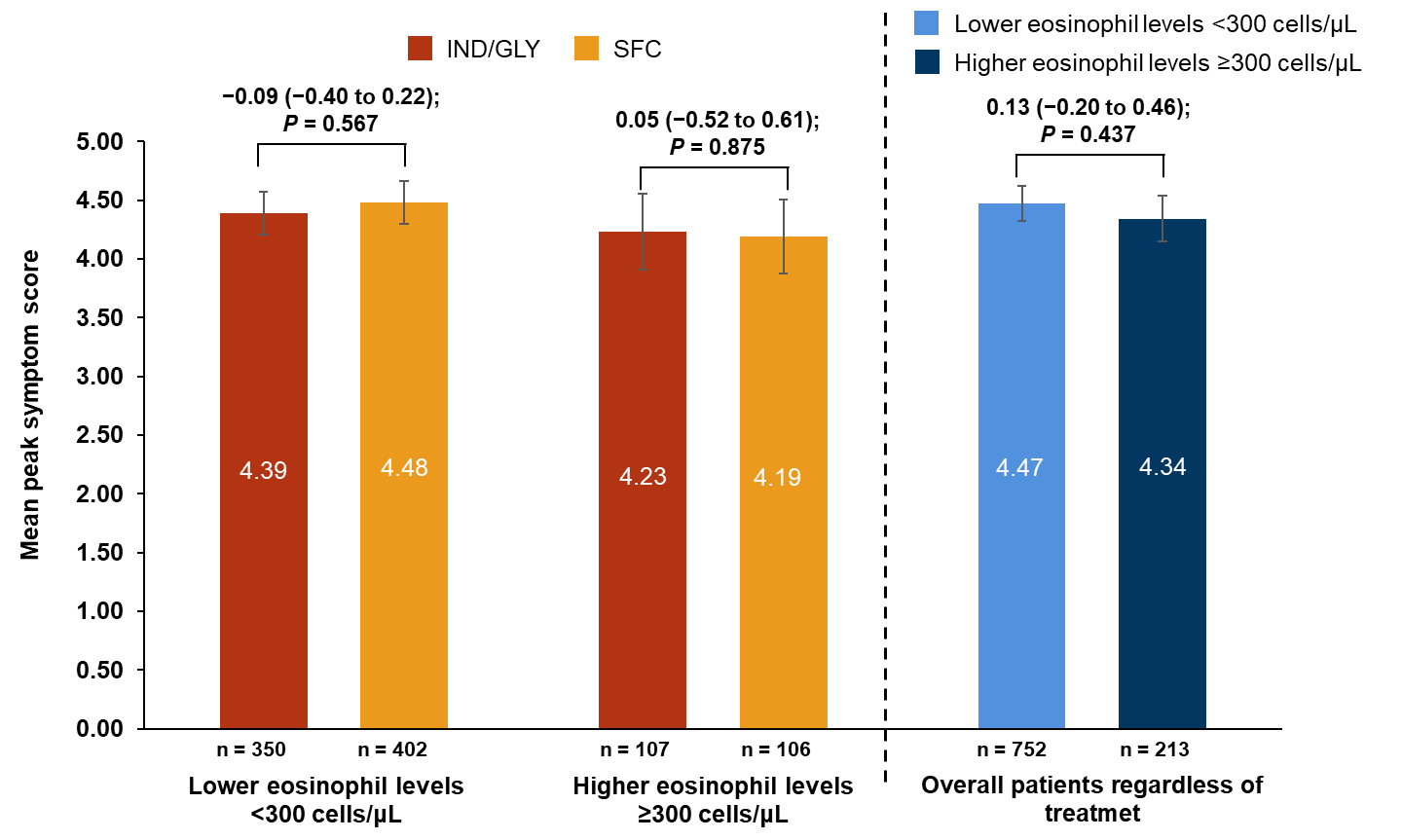** |
| --- |
| n, number of patients in each group.  In figure S9 data are presented as LSM ± SE. Differences between groups are presented as mean difference or LSM difference with 95% CI.  IND/GLY, indacaterol/glycopyrronium 110/50 µg once daily; LSM, least squares mean; SFC, salmeterol/fluticasone 50/500 µg twice daily |

**Figure S10. Annualised rate of moderate or severe exacerbations by smoking status**

| 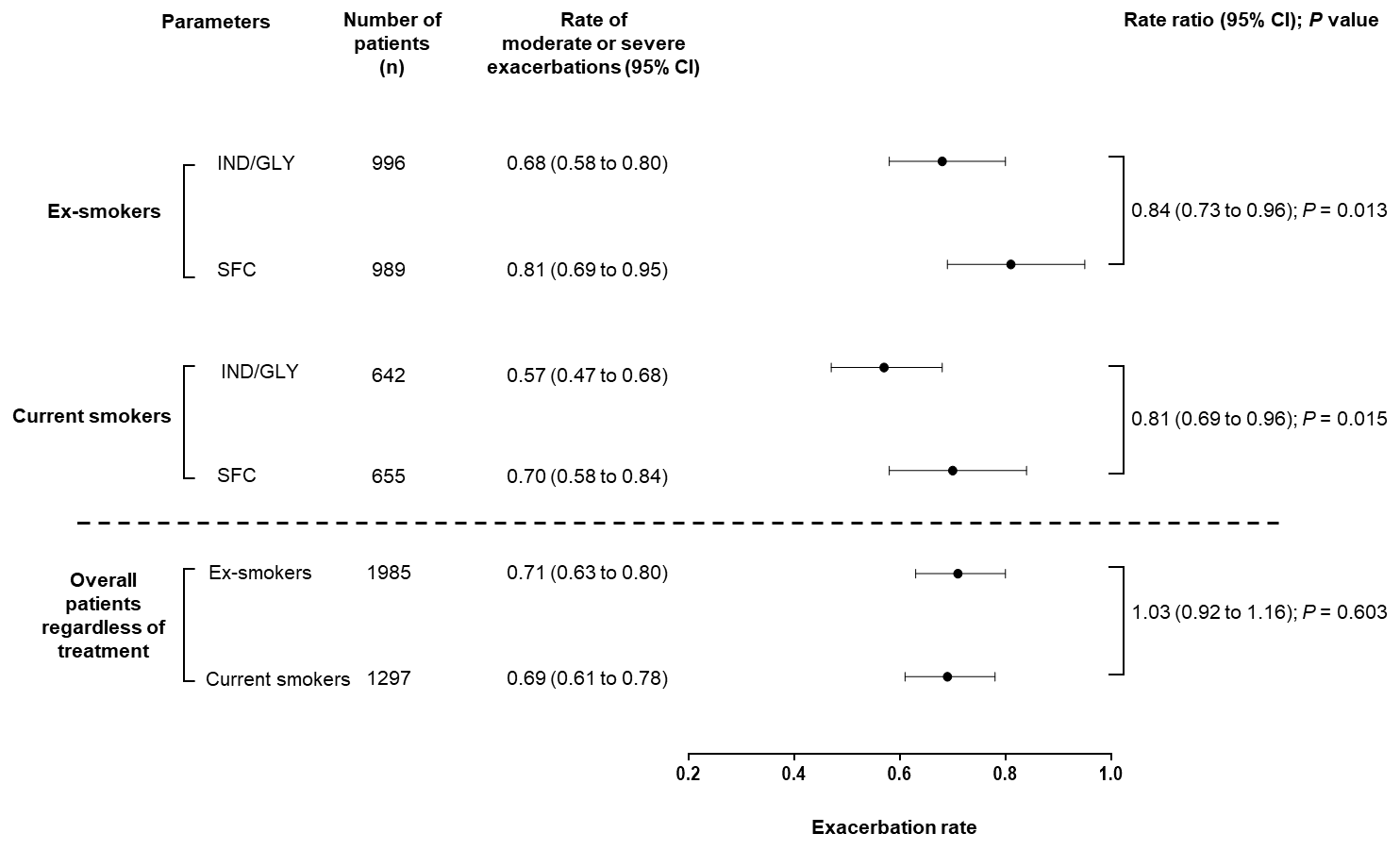 |
| --- |
| n, number of patients assessed in this analysis.  eDiary, electronic diary; IND/GLY, indacaterol/glycopyrronium 110/50 µg once daily; SFC, salmeterol/fluticasone 50/500 µg twice daily |

**Section S1:**

**Approvals**

The FLAME study protocol and all amendments were reviewed by an Independent Ethics Committee or Institutional Review Board for each center in each country (Japan, Korea, Latvia, Lithuania, Mexico, Netherlands, Norway, Philippines, Poland, Portugal, Romania, Russia, Slovakia, South Africa, Spain, Sweden, Taiwan, Turkey, United Kingdom, Thailand, Croatia, Serbia, Argentina, Austria, Belgium, Bulgaria, Canada, Chile, China, Colombia, Czech Republic, Denmark, Estonia, Finland, France, Germany, Greece, Guatemala, Hong Kong, Hungary, Iceland, India, Italy).

**SGRQ-C permission document**

**Please click on the figure to open the document.**

**References:**

1. Wedzicha JA, Banerji D, Chapman KR, et al. Indacaterol-glycopyrronium versus salmeterol-fluticasone for COPD. N Engl J Med. 2016;374(23):2222-2234.
2. Mackay AJ, Kostikas K, Murray L, Martinez FJ, Miravitlles M, Donaldson G, Banerji D, Patalano F, Wedzicha JA. Patient-reported outcomes for the detection, quantification, and evaluation of chronic obstructive pulmonary disease exacerbations. Am J Respir Crit Care Med. 2018;198(6):730-738.
